# Supplementary material for: A Shigella sonnei clone with extensive drug resistance associated with waterborne outbreaks in China
Source: Nat Commun. 2022 Nov 30;13:7365. doi: 10.1038/s41467-022-35136-1 (PMC9709761; doi:10.1038/s41467-022-35136-1)

A *Shigella sonnei* clone with extensive drug resistance associated with waterborne outbreaks in China

Supplementary Figures

- Figure S1. S1–PFGE and Southern blotting analysis of the *mcr-1*-, $bla_{CTX\text{-}M\text{-}14}$, and *mphA*-positive plasmids
- Figure S2. Annual precipitation and flood-affected areas in Guangxi and Anhui Provinces from 2011 to 2020
- Figure S3. Box plot representing the antimicrobial resistance gene content among different groups
- Figure S4. Distribution, related resistance genes, and structures of integrons
- Figure S5. Distribution of virulence-encoding genes with different functions
- Figure S6. Pangenome analysis of global *S. sonnei* isolates
- Figure S7. Temporal distribution of cases from the outbreaks

Supplementary Tables

- Table S1. Characteristics of the patients from the six outbreaks
- Table S2. Antimicrobial resistance of the Chinese *S. sonnei* isolates including the outbreak strains
- Table S3. The genome-sequenced *S. sonnei* isolates used in this study (a separate EXCEL file)
- Table S4. The plasmids identified in this study

- Table S5. The SNPs detected associated with cef^{R}azi^{R} isolates especially the outbreak strains
- Table S6. The SNPs associated with virulence-encoding genes
- Table S7. The accessory genes identified associated with cef^{R}azi^{R} isolates, especially the outbreak strains
- Table S8. The primers used in this study

Supplementary Figures

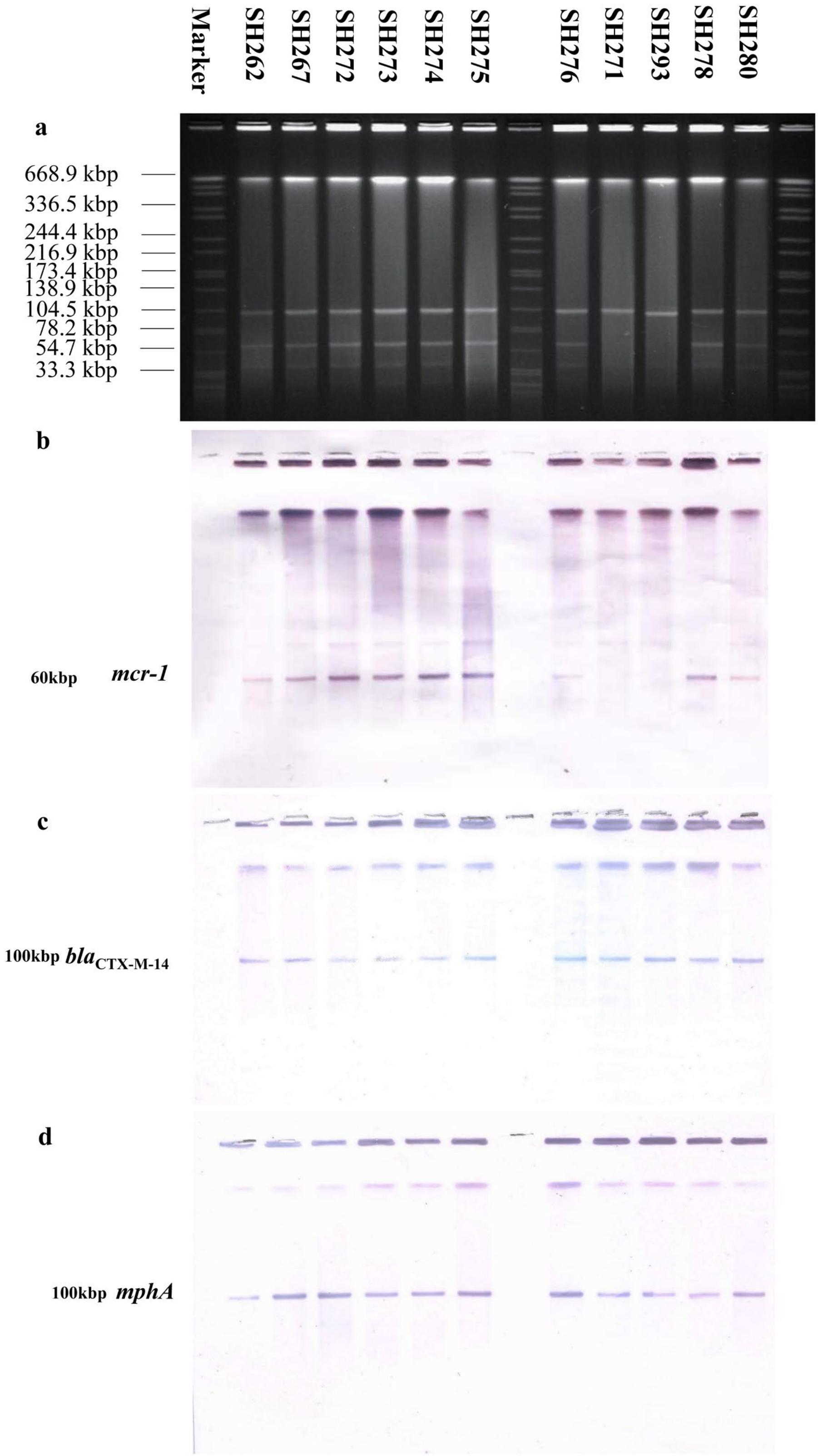

Figure S1. S1–PFGE and Southern blotting analysis of the *mcr-1*-, *bla*$_{\text{CTX-M-14}}$, and *mphA*-positive plasmids. Eleven representative isolates, including ten from patients and one from tap water, were selected for S1-PFGE and Southern hybridization analysis. The universal standard strain *Salmonella braenderup* H9812 digested with XbaI was used as the size marker for the PFGE and southern. The size of the Nylon membrane was the same as the PFGE. All experiments were independently repeated twice. **a** S1 nuclease plasmid profile obtained by PFGE. **b** Southern hybridization for the *mcr-1* gene. **c** Southern hybridization for the *bla*$_{\text{CTX-M-14}}$ gene. **d** Southern hybridization for the *mphA* gene. Source data are provided as a Source Data file and are provided at the end of this file. S1–PFGE S1 nuclease pulsed-field gel electrophoresis.

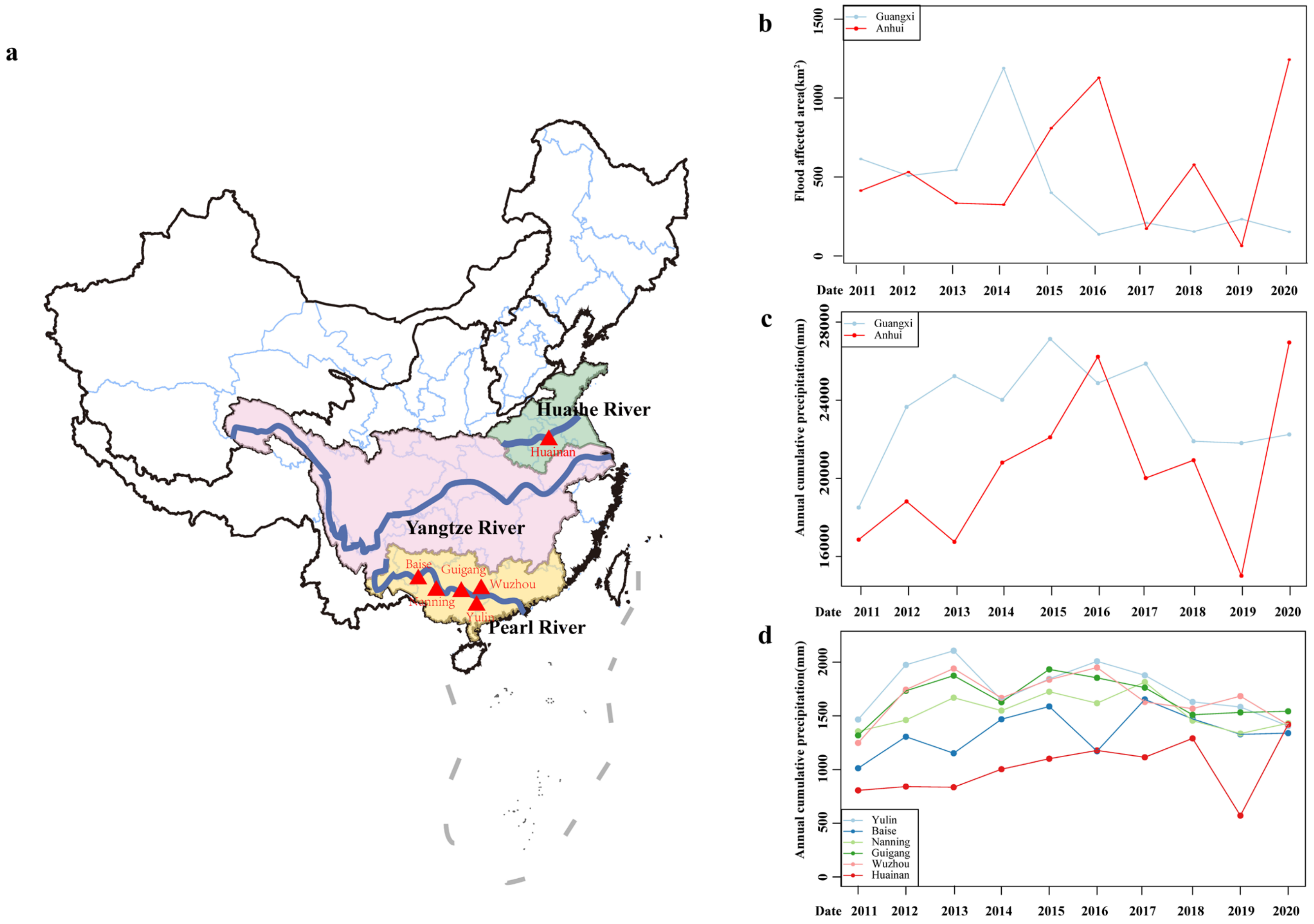

Figure S2. Annual precipitation and flood-affected areas in Guangxi and Anhui Provinces from 2011 to 2020. a shows the outbreak locations and the major rivers flowing through the regions. **b** shows the flood-affected areas in Anhui and Guangxi Provinces of China from 2011 to 2020. **c** shows the annual cumulative precipitation in Anhui and Guangxi Provinces of China from 2011 to 2020. **d** shows the annual cumulative precipitation in six outbreak locations from 2011 to 2020. The map was created using publicly available data (https://www.webmap.cn/main.do?method=index). Source data are provided as a Source Data file.

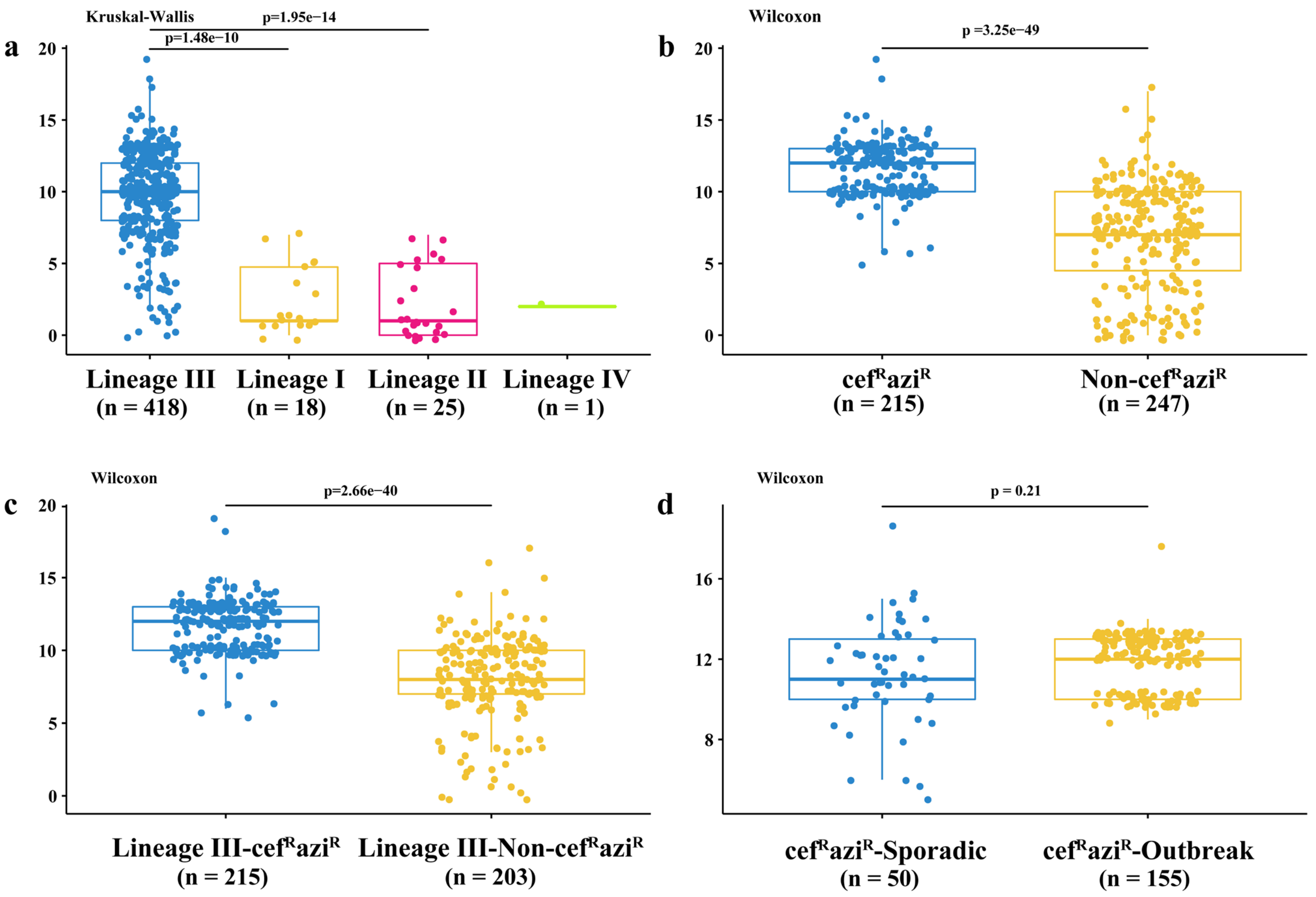

Figure S3. Box plot representing the antimicrobial resistance gene content among different groups. a shows that lineage III strains carry more AMR genes than other lineages. **b** and **c** show that the cef^{R}azi^{R} strains carry more AMR genes than non-cef^{R}azi^{R} strains as a whole or in lineage III. **d** indicates that the gene number shows no significant difference in the outbreak cef^{R}azi^{R} and sporadic cef^{R}azi^{R} strains. Centerline, median; box limits, upper and lower quartiles; whiskers, 1.5x interquartile range; points, individual data. All statistical tests were two-sided. Source data are provided as a Source Data file. cef^{R}azi^{R} coresistance to ceftriaxone and azithromycin, AMR antimicrobial resistance.

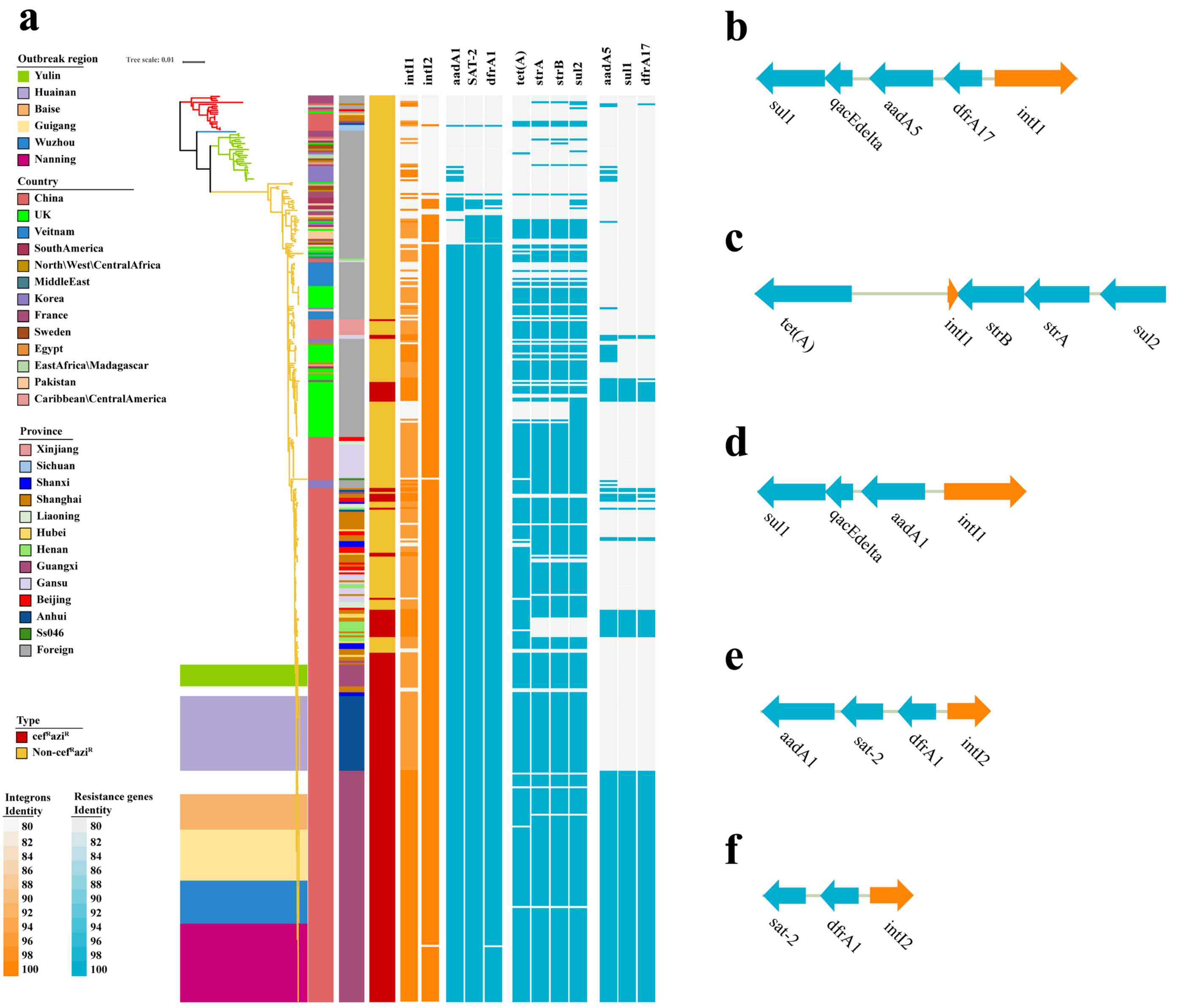

Figure S4. Distribution, related resistance genes, and structures of integrons. a shows 10 resistance genes associated with integrons. **b** to **f** show the structures of the five integrons. Source data are provided as a Source Data file. cef^{R}azi^R coresistance to ceftriaxone and azithromycin.

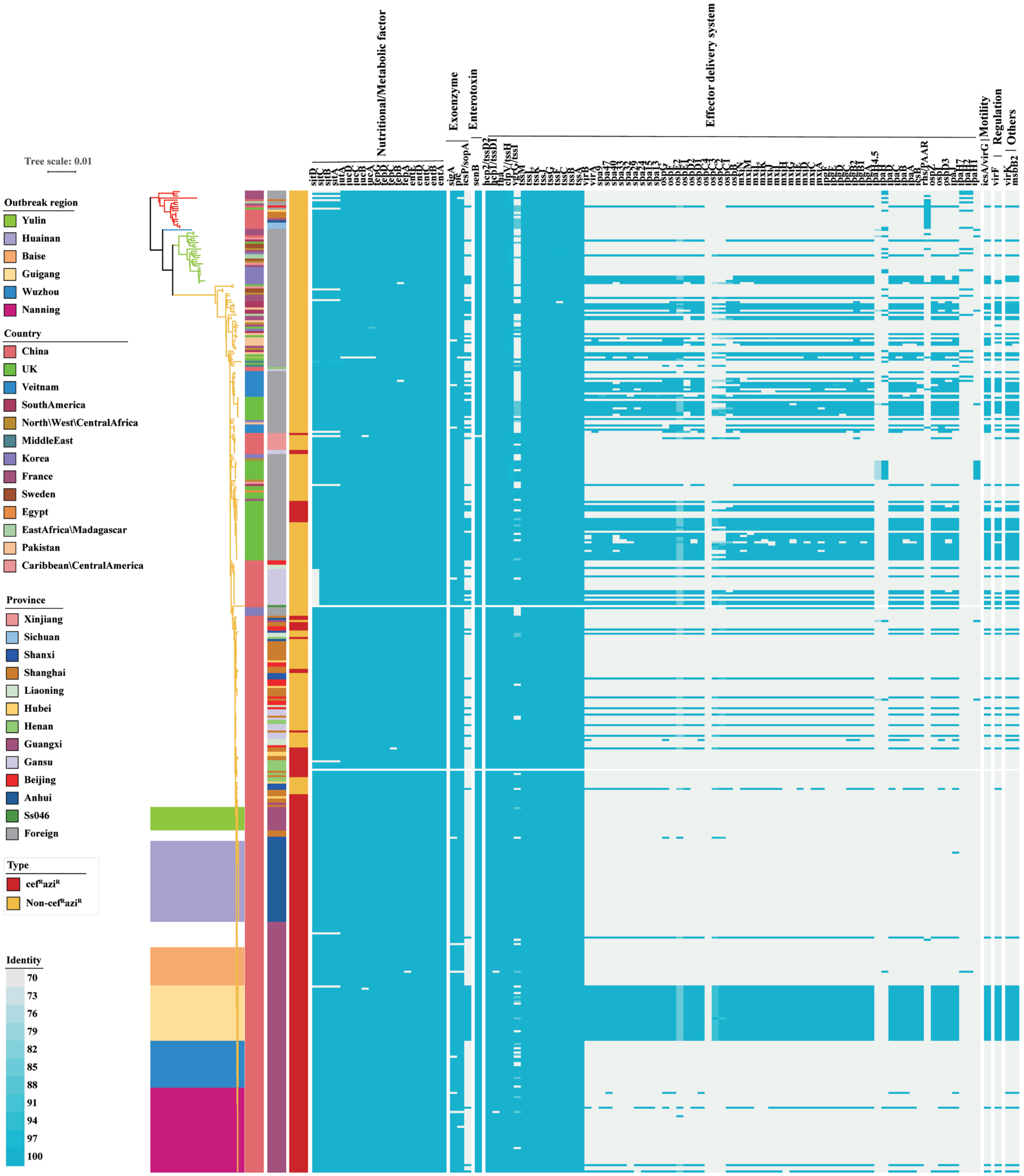

Figure S5. Distribution of virulence-encoding genes with different functions. The exoenzyme, effector delivery system, motility, regulation, and two other function genes were absent in most of the strains. There were no significant associations between the genes and the cef^{R}azi^R strains. Source data are provided as a Source Data file. cef^{R}azi^R coresistance to ceftriaxone and azithromycin.

a

b

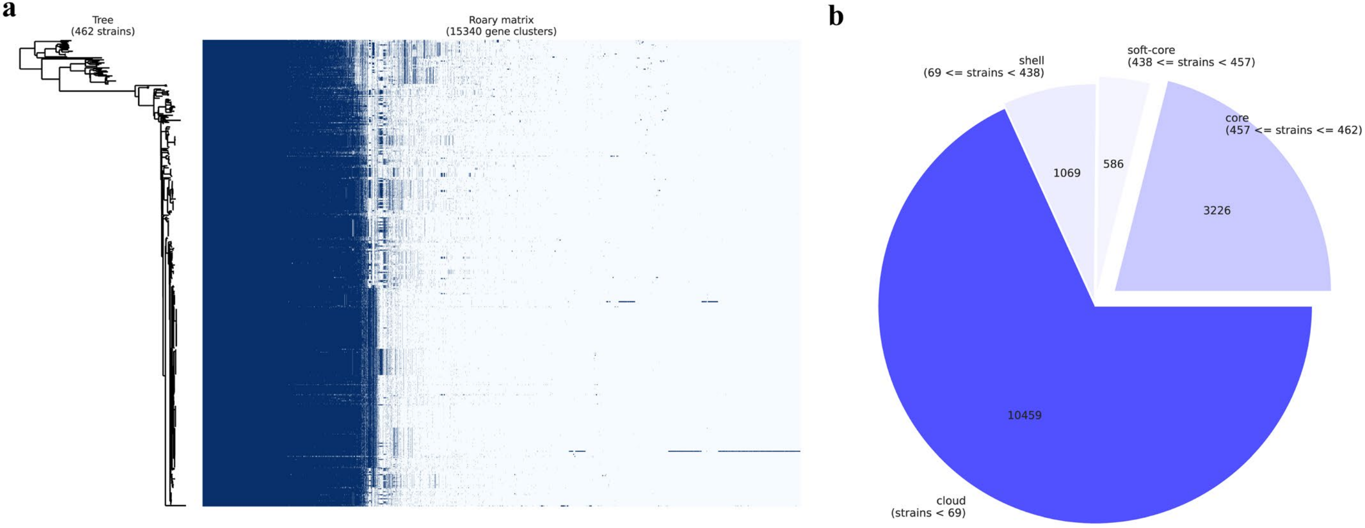

Figure S6. Pangenome analysis of global *S. sonnei* isolates. A total of 15,340 genes were identified by pangenome analysis based on the 462 isolates, 12,114 of which were assigned to the accessory genome. **a** shows a heatmap displaying the distribution of the pangenome among isolates. Dark blue indicates the presence of a gene, and light blue indicates the absence of a gene. **b** shows the proportion of core and accessory genes. Source data are provided as a Source Data file.

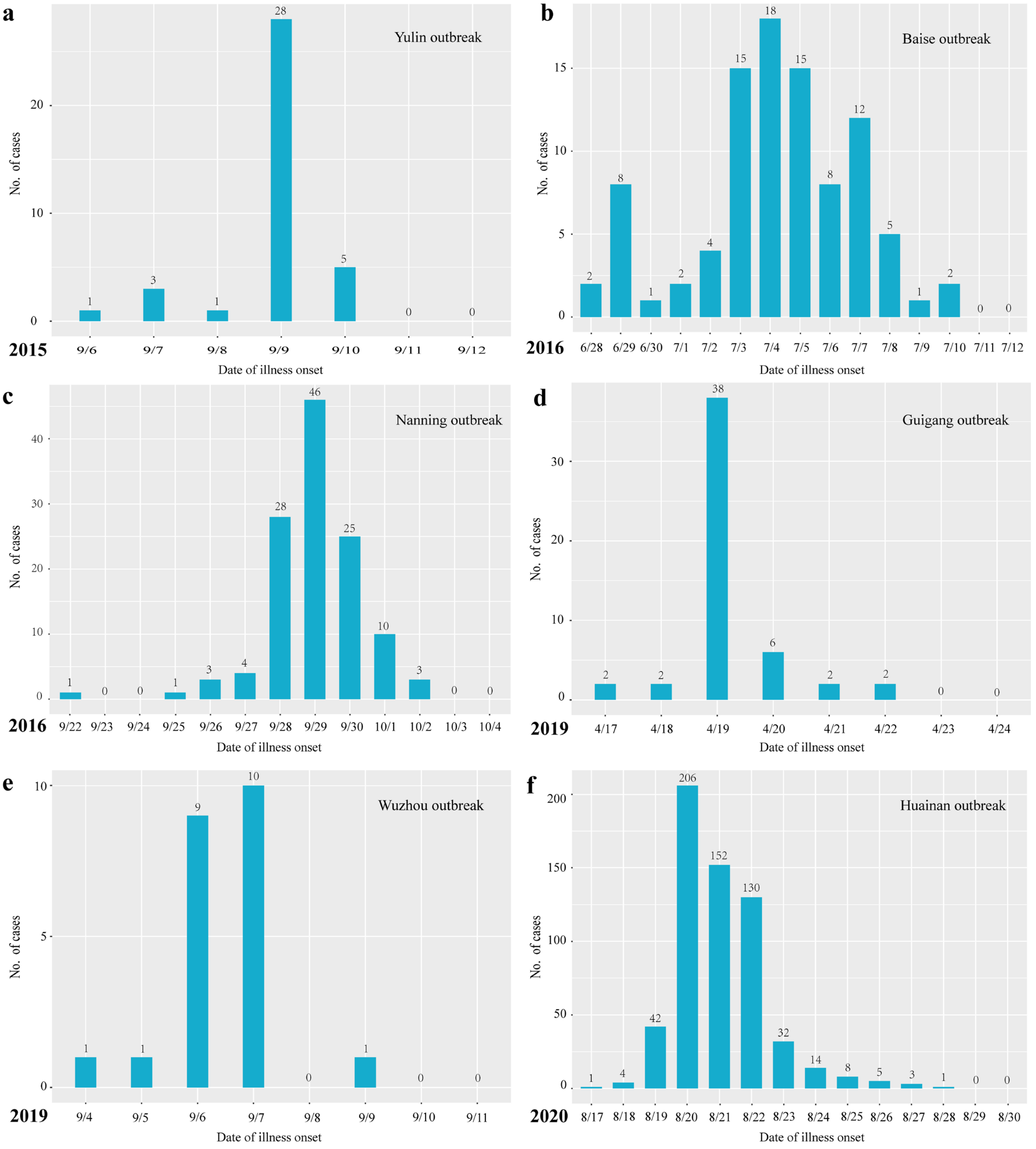

Figure S7. Temporal distribution of cases from the outbreaks. a to **f** show the temporal distribution of the cases from the six outbreaks, respectively. Source data are provided as a Source Data file.

Supplementary Tables

Table S1. Characteristics of the patients from the six outbreaks

Characteristics	Yulin Outbreak (N = 38)	Baise Outbreak (N = 93)	Nanning Outbreak (N = 121)	Guigang Outbreak (N = 52)	Wuzhou Outreak (N = 22)	Huainan Outbreak (n = 107)	Total (N = 433)
Geographical latitude	Southern China	Southern China	Southern China	Southern China	Southern China	Eastern China	Southern and Eastern China
Province	Guangxi	Guangxi	Guangxi	Guangxi	Guangxi	Anhui	Guangxi and Anhui
Outbreak location	Town	Town	Town	Village	Town	Town	Village and towns
Outbreak unit	Kindergarten	Primary school	Middle school	Kindergarten	Middle school	Kindergarten and Primary school	Kindergartens and schools
Year	2015	2016	2016	2019	2019	2020	2015-2020
Occupation, no. (%)							
Preschool children	38 (100)	35 (37.6)	0	52 (100)	0	25 (23.4)	150 (34.6)
Primary school student	0	51 (54.8)	0	0	2 (9.1)	59 (55.1)	112 (25.9)
Junior high school student	0	0	119 (98.3)	0	19 (86.4)	0	138 (31.9)
Teaching staff	0	2 (2.2)	2 (1.7)	0	1 (4.5)	0	5 (1.2)
Farmer	0	5 (5.4)	0	0	0	23 (21.5)	28 (6.5)
Age, no. (%)							
≤ 3 yr	12 (31.6)	16 (17.2)	0	6 (11.5)	0	0	34 (7.9)
4-6 yr	26 (68.4)	19 (20.4)	0	46 (88.5)	0	25 (23.4)	116 (26.8)
7-14 yr	0	51 (54.8)	119 (98.3)	0	21 (95.5)	59 (55.1)	250 (57.7)
> 14 yr	0	7 (7.5)	2 (1.7)	0	1 (4.5)	23 (21.5)	33 (7.6)

Male sex, no. (%)	23 (61)	40 (43)	45 (37)	33 (63)	14 (64)	54 (50)	209 (48.3)
Symptoms, no. (%)							
Diarrhea	38 (100)	93 (100)	121 (100)	52 (100)	22 (100)	107 (100)	433 (100)
Fever	37 (97.4)	89 (95.7)	113 (93.4)	47 (90.4)	20 (90.9)	101 (94.4)	407 (94.0)
Abdominal pain	30 (57.7)	55 (59.1)	84 (69.4)	52 (100)	16 (72.7)	67 (62.6)	304 (70.2)
Vomitting	27 (71.1)	72 (77.4)	82 (67.8)	29 (55.8)	11 (50.0)	72 (67.3)	293 (67.7)
Rectal Tenesmus	26 (68.4)	60 (64.5)	62 (51.2)	35 (67.3)	35 (67.3)	0	218 (50.3)
Nausea	10 (26.3)	25 (26.9)	34 (28.1)	1 (1.9)	3 (13.6)	23 (21.5)	96 (22.2)
Dizziness	0	3 (3.2)	0	1 (1.9)	0	3 (2.8)	7 (1.6)

Table S2. Antimicrobial resistance of the Chinese *S. sonnei* isolates including the outbreak strains

Antimicrobials	Antimicrobial resistance (%)								
	Outbreak							Sporadic (n = 152)	All (n = 307)
	Yulin outbreak (n = 11)	Baise outbreak (n = 18)	Nanning outbreak (n = 40)	Guigang outbreak (n = 26)	Wuzhou outreak (n = 22)	Huainan outbreak (n = 38)	Total (n = 155)		
Ceftazidime	0.0	0.0	0.0	0.0	0.0	0.0	0.0	2.6	1.3
Ceftriaxone	100.0	100.0	100.0	100.0	100.0	100.0	100.0	82.2	91.2
Imipenem	0.0	0.0	0.0	0.0	0.0	0.0	0.0	0.0	0.0
Nitrofurantoin	0.0	0.0	0.0	0.0	0.0	0.0	0.0	0.7	0.3
Piperacillin	100.0	77.8	100.0	100.0	100.0	100.0	97.4	70.4	84.0
Tetracycline	100.0	100.0	100.0	100.0	100.0	100.0	100.0	84.9	92.5
Cefepime	0.0	0.0	0.0	0.0	0.0	0.0	0.0	0.0	0.0
Cefoperazone	100.0	100.0	100.0	100.0	100.0	100.0	100.0	67.1	83.7
Cefazolin	100.0	100.0	100.0	100.0	100.0	100.0	100.0	82.2	91.2
Cefoxitin	0.0	0.0	5.0	0.0	0.0	0.0	1.3	5.3	3.3
Tobramycin	100.0	83.3	87.5	100.0	100.0	100.0	94.8	31.6	63.5
Levofloxacin	0.0	0.0	0.0	0.0	0.0	0.0	0.0	0.0	0.0
Gentamicin	100.0	100.0	100.0	100.0	100.0	100.0	100.0	62.5	81.4
Ticarcillin	100.0	100.0	100.0	100.0	100.0	100.0	100.0	84.9	92.5
Ticarcillin/clavulanic acid	0.0	0.0	5.0	0.0	0.0	0.0	1.3	14.5	7.8
Aztreonam	0.0	0.0	2.5	3.8	0.0	0.0	1.3	15.8	8.5
Ampicillin	100.0	100.0	100.0	100.0	100.0	100.0	100.0	84.2	92.2
Chloramphenicol	0.0	0.0	0.0	0.0	0.0	0.0	0.0	3.3	1.6
Trimethoprim/sulfamethoxazole	100.0	100.0	100.0	100.0	100.0	100.0	100.0	86.8	93.5

Norfloxacin	0.0	0.0	0.0	0.0	0.0	0.0	0.0	0.0	0.0
Amikacin	0.0	0.0	0.0	0.0	0.0	0.0	0.0	0.0	0.0
Azithromycin	100.0	100.0	100.0	100.0	100.0	100.0	100.0	32.9	66.8
Colistin	0.0	0.0	75.0	0.0	0.0	0.0	19.4	2.0	10.7
$cef^{R}azi^{R}$	100.0	100.0	100.0	100.0	100.0	100.0	100.0	32.9	66.8
$cef^{R}azi^{R}col^{R}$	0.0	0.0	75.0	0.0	0.0	0.0	19.4	0.0	9.8

Table S3. The genome-sequenced *S. sonnei* isolates used in this study. Due to the page size limitation, Supplementary Table S3 is provided as a separate EXCEL file.

Table S4. The plasmids identified in this study

Plasmid	GenBank no.	Replicon type	Size of plasmid (bp)	Resistance gene	Isolate	Region	Year
pSH262-1	MG299127	IncI2	63066	mcr-1	SH262	Guangxi Province	2016
pSH267-1	MG299129	IncI2	63066	mcr-1	SH267	Guangxi Province	2016
pSH272-1	MG299132	IncI2	62958	mcr-1	SH272	Guangxi Province	2016
pSH273-1	MG299134	IncI2	63066	mcr-1	SH273	Guangxi Province	2016
pSH274-1	MG299136	IncI2	63066	mcr-1	SH274	Guangxi Province	2016
pSH275-1	MG299138	IncI2	63066	mcr-1	SH275	Guangxi Province	2016
pSH276-1	MG299140	IncI2	62958	mcr-1	SH276	Guangxi Province	2016
pSH278-1	MG299142	IncI2	63066	mcr-1	SH278	Guangxi Province	2016
pSH280-1	MG299144	IncI2	63066	mcr-1	SH280	Guangxi Province	2016
pSH284-1	MG299146	IncI2	63066	mcr-1	SH284	Guangxi Province	2016
pSH286-1	MG299148	IncI2	62958	mcr-1	SH286	Guangxi Province	2016

pSH287-1	MG299150	IncI2	62958	mcr-1	SH287	Guangxi Province	2016
pSH292-1	MG299152	IncI2	62958	mcr-1	SH292	Guangxi Province	2016
pSH262-2	MG299128	IncB/O/K/Z	109845	blaCTX-M-14/mphA/aac(3)-IId/aadA5/dfrA17/sul1	SH262	Guangxi Province	2016
pSH267-2	MG299130	IncB/O/K/Z	109845	blaCTX-M-14/mphA/aac(3)-IId/aadA5/dfrA17/sul1	SH267	Guangxi Province	2016
pSH271-2	MG299131	IncB/O/K/Z	109845	blaCTX-M-14/mphA/aac(3)-IId/aadA5/dfrA17/sul1	SH271	Guangxi Province	2016
pSH272-2	MG299133	IncB/O/K/Z	109845	blaCTX-M-14/mphA/aac(3)-IId/aadA5/dfrA17/sul1	SH272	Guangxi Province	2016
pSH273-2	MG299135	IncB/O/K/Z	109845	blaCTX-M-14/mphA/aac(3)-IId/aadA5/dfrA17/sul1	SH273	Guangxi Province	2016
pSH274-2	MG299137	IncB/O/K/Z	109845	blaCTX-M-14/mphA/aac(3)-IId/aadA5/dfrA17/sul1	SH274	Guangxi Province	2016
pSH275-2	MG299139	IncB/O/K/Z	109845	blaCTX-M-14/mphA/aac(3)-IId/aadA5/dfrA17/sul1	SH275	Guangxi Province	2016
pSH276-2	MG299141	IncB/O/K/Z	109845	blaCTX-M-14/mphA/aac(3)-IId/aadA5/dfrA17/sul1	SH276	Guangxi Province	2016
pSH278-2	MG299143	IncB/O/K/Z	109845	blaCTX-M-14/mphA/aac(3)-IId/aadA5/dfrA17/sul1	SH278	Guangxi Province	2016
pSH280-2	MG299145	IncB/O/K/Z	109845	blaCTX-M-14/mphA/aac(3)-IId/aadA5/dfrA17/sul1	SH280	Guangxi Province	2016
pSH284-2	MG299147	IncB/O/K/Z	109845	blaCTX-M-14/mphA/aac(3)-IId/aadA5/dfrA17/sul1	SH284	Guangxi Province	2016

pSH286-2	MG299149	IncB/O/K/Z	109845	blaCTX-M-14/mphA/aac(3)-IId/aadA5/dfrA17/sul1	SH286	Guangxi Province	2016
pSH287-2	MG299151	IncB/O/K/Z	109845	blaCTX-M-14/mphA/aac(3)-IId/aadA5/dfrA17/sul1	SH287	Guangxi Province	2016
pSH292-2	MG299153	IncB/O/K/Z	109845	blaCTX-M-14/mphA/aac(3)-IId/aadA5/dfrA17/sul1	SH292	Guangxi Province	2016
pSH13D178-1		IncB/O/K/Z	108915	ermB/mphA/aadA5/dfrA17/sul1	SH13D178	Shanghai City	2013
pSH13D178-2		IncFII	96612	blaTEM-1/mphA/aac(3)-IId/aadA5/dfrA17/sul1	SH13D178	Shanghai City	2013
pSH13D178-3		IncI1-(Alpha)	81100	blaCMY-2	SH13D178	Shanghai City	2013
pSH11sh418-1		IncI1-(Alpha)	113793	blaCTX-M-14/blaTEM-1/aac(3)-IId	SH11sh418	Shanghai City	2011

Table S5. The SNPs detected associated with $cef^{R}azi^{R}$ isolates especially the outbreak strains(Chi-squared test, adjusted by FDR correction)

SNP position[a]	SNP	Mutation type	gene	start	end	product	P value (Outbreak strains associated)[b]	P value ($cef^{R}azi^{R}$ strains associated)[b]	COG	Code	Gene in COG
148125	C>T	Samesense mutation	gcd	147811	150201	glucose dehydrogenase	6.16E-84	3.92E-94			
172616	G>A	Samesense mutation	pcnB	171926	173290	poly(A) polymerase I	4.88E-77	2.20E-98			
561422	C>T	Samesense mutation	ybdE	559031	562174	putative inner membrane component for iron	2.61E-154	2.33E-136			
720375	C>T	Samesense mutation	sucA	719395	722196	2-oxoglutarate dehydrogenase decarboxylase component	1.06E-98	6.50E-81			
824594	A>C	Samesense mutation	ybiB	824118	825080	putative enzyme	2.02E-74	2.22E-94			
844182	C>T	Samesense mutation	ybiR	843811	844929	conserved hypothetical protein	2.02E-74	2.22E-94			
947726	T>C	Samesense mutation	ftsK	947043	951110	cell division protein	2.02E-74	2.22E-94			
1207876	C>T	Samesense mutation	ndh	1207384	1208688	respiratory NADH dehydrogenase	8.09E-132	7.28E-151			
1213343	C>T	Samesense mutation	mfd	1211828	1215274	transcription-repair coupling factor	2.02E-74	2.22E-94			
2300159	A>G	Samesense mutation	yohF	2299442	2300203	putative oxidoreductase	7.40E-62	3.28E-75			
2373264	A>G	Samesense mutation	napB	2372869	2373339	cytochrome c-type protein	7.40E-62	3.28E-75			
2465186	C>A	Samesense mutation	nuoL	2464205	2466046	NADH dehydrogenase I chain L	2.61E-154	2.33E-136			
2639389	A>G	Samesense mutation	zipA	2638840	2639826	cell division protein	2.02E-74	2.22E-94			
2643003	A>G	Samesense mutation	ptsI	2642659	2644386	PEP-protein phosphotransferase system enzyme I	7.40E-62	3.28E-75			
2745444	G>A	Samesense mutation	yfgA	2745373	2746386	putative membrane protein	1.06E-98	6.50E-81			
3055920	A>T	Samesense mutation	cysI	3054225	3055937	sulfite reductase, alpha subunit	7.40E-62	3.28E-75			
3655517	G>A	Samesense mutation	yhfC	3654567	3655748	putative transport	4.88E-77	2.20E-98			
3821395	G>A	Samesense mutation	recF	3821059	3822132	ssDNA and dsDNA binding protein	4.88E-77	2.20E-98			
3885819	G>A	Samesense mutation	zntA	3883720	3885918	zinc-transporting ATPase	4.88E-77	2.20E-98			
4189282	G>A	Samesense mutation	yifM	4188215	4189294	Uncharacterized conserved protein	7.87E-64				
4226472	A>G	Samesense mutation	metR	4225953	4226906	regulator for metE and metH	7.40E-62	3.28E-75			
4491950	T>C	Samesense mutation	tyrB	4491537	4492730	tyrosine aminotransferase, tyrosine repressible	2.02E-74	2.22E-94			
4624346	A>G	Samesense mutation	mutL	4624260	4626107	enzyme in methyl-directed mismatch repair	8.22E-82	4.33E-91			
4634057	C>T	Samesense mutation	vacB	4633461	4635944	putative enzyme	4.88E-77	2.20E-98			
3194910	G>A	Nonsense mutation	SSON_3035	3193619	3196474	putative dehydrogenase	1.06E-98	6.50E-81	COG1529	C	CoxL
148674	A>G	Missense mutation	gcd	147811	150201	glucose dehydrogenase		1.27E-53	COG4993	G	Gcd
230238	T>C	Missense mutation	yaeO	230038	230298	conserved hypothetical protein	2.02E-74	2.22E-94	COG4568	K	Rof
300237	C>T	Missense mutation	fhiA	299468	301207	flagellar biosynthesis	4.88E-77	2.20E-98	COG1298	NU	FhlA
370713	C>T	Missense mutation	tauB	370289	371056	taurine ATP-binding component of a transport system	3.17E-100	6.50E-81	COG4525	P	TauB
391136	C>T	Missense mutation	aroM	390817	391494	protein of aro operon, regulated by aroR	8.22E-82	4.33E-91			
441036	A>G	Missense mutation	ampG	440867	442342	regulates beta-lactamase synthesis	4.88E-77	2.20E-98	COG0477	GEPR	ProP
562166	C>T	Missense mutation	ybdE	559031	562174	putative inner membrane component for iron	1.73E-158	2.97E-141	COG3696	P	CusA
667248	C>T	Missense mutation	nagE	666825	668771	PTS system, N-acetylglucosamine-specific enzyme	4.88E-77	2.20E-98	COG1263	G	PtsG1
727336	T>C	Missense mutation	hrsA	726492	728468	protein modification enzyme, induction of ompC	7.40E-62	3.28E-75	COG1299	G	FrwC
1201878	C>T	Missense mutation	fhuE	1200653	1202842	outer membrane receptor for ferric iron uptake		1.27E-53	COG4773	P	FhuE
1527239	C>T	Missense mutation	SSON_1453	1526567	1528003	conserved hypothetical protein	2.02E-74	2.22E-94			
1603227	C>A	Missense mutation	ydgO	1603150	1604208	conserved hypothetical protein	3.17E-100	6.50E-81	COG4658	C	RnfD
2056926	A>G	Missense mutation	SSON_1954	2056895	2057113	conserved hypothetical protein	4.88E-77	2.20E-98			
2169250	T>C	Missense mutation	pduS	2168853	2169755	putative propanediol utilization protein	8.09E-132	7.28E-151	COG4656	C	RnfC
2287864	C>G	Missense mutation	yehV	2287841	2288488	putative transcriptional regulator	8.09E-132	7.28E-151	COG0789	K	SoxR
2411350	A>G	Missense mutation	gyrA	2408970	2411597	DNA gyrase subunit A	5.97E-52	3.56E-60	COG0188	L	GyrA
2492128	T>A	Missense mutation	yfcG	2491612	2492259	putative S-transferase	4.88E-77	2.20E-98	COG0625	O	GstA
2524499	C>T	Missense mutation	yfcU	2524198	2526840	PapC-like porin protein	4.88E-77	2.20E-98	COG3188	NU	FimD
2704351	C>A	Missense mutation	hyfB	2703871	2705889	hydrogenase 4 membrane subunit	2.02E-74	2.22E-94	COG0651	CP	HyfB
3013077	T>G	Missense mutation	hycA	3012841	3013302	transcriptional repression of hyc and hyp	2.24E-78	1.47E-95			
3032159	G>T	Missense mutation	nlpD	3031258	3032397	lipoprotein	6.16E-84	3.92E-94	COG0739	M	NlpD
3083989	G>A	Missense mutation	ygcA	3083426	3084727	putative enzyme	2.24E-78	1.47E-95	COG2265	J	TrmA
3317052	A>G	Missense mutation	ygiRQ	3315254	3317473	Fe-S oxidoreductase	2.02E-74	2.22E-94	COG1032	C	YgiQ
3371683	G>C	Missense mutation	yqjH	3371397	3372161	conserved hypothetical protein	6.16E-84	3.92E-94	COG2375	P	ViuB
3938553	G>A	Missense mutation	gltS	3938436	3939641	glutamate transport	2.61E-154	2.33E-136	COG0786	E	GltS
3952595	G>A	Missense mutation	recG	3939810	3941891	DNA helicase	2.24E-78	1.47E-95	COG1200	LK	RecG
4237501	C>T	Missense mutation	yigP	4237068	4237673	conserved hypothetical protein	7.40E-62	3.28E-75			
4366788	T>A	Missense mutation	frwD	4366778	4367119	PTS system fructose-like IIB component 2	8.09E-132	7.28E-151	COG1445	G	FrwB
4438780	A>G	Missense mutation	iclR	4438274	4439137	repressor of aceBA operon	2.02E-74	2.22E-94	COG1414	K	IclR
4620189	T>G	Missense mutation	yjeS	4619776	4620915	conserved hypothetical protein	2.61E-154	2.33E-136	COG1600	C	QueG
4655680	C>T	Missense mutation	ytfB	4655096	4655770	conserved hypothetical protein	2.61E-154	2.33E-136	COG3061	M	OapA
4666728	G>A	Missense mutation	ytfK	4666555	4666800	conserved hypothetical protein	2.24E-78	1.47E-95			
819559	T>C	Intergenic region					2.02E-74	2.22E-94			
1959401	G>A	Intergenic region					4.88E-77	2.20E-98			
3018039	T>G	Intergenic region					2.02E-74	2.22E-94			
3851577	A>G	Intergenic region					4.88E-77	2.20E-98			

[a]SNPs were detected by mapping to the reference genome *S. sonnei* Ss046 (accession NC_007384); [b]One-sided chi-square tests were performed and P values were adjusted with FDR correction.

Table S6. The SNPs associated with virulence-encoding genes

Chrom	Position	Ref	Alt	NT pos	AA pos	Effect	Gene
CP000038	588479	C	T	398/747	133/248	missense variant c.398C>T p.Ala133Val	entA
CP000038	585275	C	T	860/1176	287/391	missense variant c.860C>T p.Ser287Leu	entC
CP000038	569125	C	T	409/630	137/209	missense variant c.409G>A p.Ala137Thr	entD
CP000038	569030	A	T	504/630	168/209	synonymous variant c.504T>A p.Gly168Gly	entD
CP000038	586449	T	C	849/1611	283/536	synonymous variant c.849T>C p.Val283Val	entE
CP000038	573908	G	A	307/3882	103/1293	missense variant c.307G>A p.Ala103Thr	entF
CP000038	576620	C	T	3019/3882	1007/1293	synonymous variant c.3019C>T p.Leu1007Leu	entF
CP000038	576121	C	T	2520/3882	840/1293	synonymous variant c.2520C>T p.Asp840Asp	entF
CP000038	575328	G	A	1727/3882	576/1293	missense variant c.1727G>A p.Cys576Tyr	entF
CP000038	576954	C	T	3353/3882	1118/1293	missense variant c.3353C>T p.Ala1118Val	entF
CP000038	573843	A	C	242/3882	81/1293	missense variant c.242A>C p.Gln81Pro	entF
CP000038	577429	T	A	3828/3882	1276/1293	synonymous variant c.3828T>A p.Ser1276Ser	entF
CP000038	577032	C	T	3431/3882	1144/1293	missense variant c.3431C>T p.Ala1144Val	entF
CP000038	576900	C	T	3299/3882	1100/1293	missense variant c.3299C>T p.Pro1100Leu	entF
CP000038	574693	G	A	1092/3882	364/1293	synonymous variant c.1092G>A p.Gln364Gln	entF
CP000038	571467	C	T	473/2241	158/746	missense variant c.473G>A p.Gly158Asp	fepA
CP000038	571232	G	T	708/2241	236/746	synonymous variant c.708C>A p.Ala236Ala	fepA
CP000038	569890	T	C	2050/2241	684/746	missense variant c.2050A>G p.Thr684Ala	fepA
CP000038	569981	C	T	1959/2241	653/746	synonymous variant c.1959G>A p.Gln653Gln	fepA
CP000038	569919	C	T	2021/2241	674/746	missense variant c.2021G>A p.Ser674Asn	fepA
CP000038	583136	C	T	906/966	302/321	synonymous variant c.906G>A p.Leu302Leu	fepB
CP000038	583475	T	G	567/966	189/321	missense variant c.567A>C p.Leu189Phe	fepB
CP000038	583325	C	T	717/966	239/321	synonymous variant c.717G>A p.Gln239Gln	fepB
CP000038	583315	G	A	727/966	243/321	missense variant c.727C>T p.His243Tyr	fepB
CP000038	579258	C	T	387/816	129/271	synonymous variant c.387G>A p.Gln129Gln	fepC
CP000038	579407	C	A	238/816	80/271	missense variant c.238G>T p.Ala80Ser	fepC
CP000038	579054	C	T	591/816	197/271	synonymous variant c.591G>A p.Leu197Leu	fepC
CP000038	579600	C	T	45/816	15/271	synonymous variant c.45G>A p.Leu15Leu	fepC

CP000038	578978	C	T	667/816	223/271	missense variant c.667G>A p.Val223Ile	fepC
CP000038	581008	A	G	627/1005	209/334	synonymous variant c.627T>C p.Thr209Thr	fepD
CP000038	581014	T	C	621/1005	207/334	synonymous variant c.621A>G p.Gly207Gly	fepD
CP000038	581460	C	T	175/1005	59/334	missense variant c.175G>A p.Ala59Thr	fepD
CP000038	580675	C	T	960/1005	320/334	synonymous variant c.960G>A p.Pro320Pro	fepD
CP000038	578686	C	G	988/1134	330/377	missense variant c.988C>G p.Pro330Ala	fepE
CP000038	578264	G	T	566/1134	189/377	missense variant c.566G>T p.Ser189Ile	fepE
CP000038	577867	A	C	169/1134	57/377	missense variant c.169A>C p.Ile57Leu	fepE
CP000038	578295	G	A	597/1134	199/377	synonymous variant c.597G>A p.Val199Val	fepE
CP000038	578291	T	C	593/1134	198/377	missense variant c.593T>C p.Leu198Ser	fepE
CP000038	580249	T	G	385/993	129/330	missense variant c.385A>C p.Thr129Pro	fepG
CP000038	580067	C	T	567/993	189/330	synonymous variant c.567G>A p.Thr189Thr	fepG
CP000038	795527	A	G	411/1764	137/587	synonymous variant c.411T>C p.Cys137Cys	ipaH 1
CP000038	795504	A	T	434/1764	145/587	stop gained c.434T>A p.Leu145*	ipaH 1
CP000038	795451	G	C	487/1764	163/587	missense variant c.487C>G p.Pro163Ala	ipaH 1
CP000038	795312	T	C	626/1764	209/587	missense variant c.626A>G p.Asn209Ser	ipaH 1
CP000038	795528	C	T	410/1764	137/587	missense variant c.410G>A p.Cys137Tyr	ipaH 1
CP000038	795149	C	A	789/1764	263/587	synonymous variant c.789G>T p.Leu263Leu	ipaH 1
CP000038	795843	T	C	95/1764	32/587	missense variant c.95A>G p.Tyr32Cys	ipaH 1
CP000038	1347573	T	C	239/1752	80/583	missense variant c.239A>G p.Glu80Gly	ipaH 2
CP000038	1347228	G	T	584/1752	195/583	missense variant c.584C>A p.Pro195His	ipaH 2
CP000038	1391329	A	C	520/1716	174/571	missense variant c.520T>G p.Phe174Val	ipaH 3
CP000038	1391809	A	G	40/1716	14/571	missense variant c.40T>C p.Ser14Pro	ipaH 3
CP000038	1391264	T	G	585/1716	195/571	synonymous variant c.585A>C p.Leu195Leu	ipaH 3
CP000038	1391845	A	C	4/1716	2/571	missense variant c.4T>G p.Ser2Ala	ipaH 3
CP000038	1391234	T	A	615/1716	205/571	synonymous variant c.615A>T p.Ser205Ser	ipaH 3
CP000038	1391716	T	C	133/1716	45/571	missense variant c.133A>G p.Asn45Asp	ipaH 3
CP000038	2282495	A	G	36/1644	12/547	synonymous variant c.36A>G p.Gly12Gly	ipaH 4
CP000038	2805291	C	T	324/1827	108/608	synonymous variant c.324C>T p.Phe108Phe	ipaH 5
CP000038	2805603	A	G	636/1827	212/608	synonymous variant c.636A>G p.Leu212Leu	ipaH 5

CP000038	2805552	A	G	585/1827	195/608	synonymous variant c.585A>G p.Leu195Leu	ipaH 5
CP000038	2805339	G	A	372/1827	124/608	synonymous variant c.372G>A p.Val124Val	ipaH 5
CP000038	2805281	G	A	314/1827	105/608	missense variant c.314G>A p.Arg105Lys	ipaH 5
CP000038	2805347	A	C	380/1827	127/608	missense variant c.380A>C p.Asn127Thr	ipaH 5
CP000038	2805089	A	C	122/1827	41/608	missense variant c.122A>C p.Glu41Ala	ipaH 5
CP000038	2805445	A	G	478/1827	160/608	missense variant c.478A>G p.Lys160Glu	ipaH 5
CP000038	2805966	T	A	999/1827	333/608	synonymous variant c.999T>A p.Val333Val	ipaH 5
CP000038	3767718	C	T	169/1725	57/574	missense variant c.169C>T p.Pro57Ser	iucA
CP000038	3770057	C	T	783/948	261/315	synonymous variant c.783C>T p.Pro261Pro	iucB
CP000038	3769348	G	T	74/948	25/315	missense variant c.74G>T p.Gly25Val	iucB
CP000038	3771388	C	T	1167/1743	389/580	synonymous variant c.1167C>T p.Ser389Ser	iucC
CP000038	3771250	C	T	1029/1743	343/580	synonymous variant c.1029C>T p.Tyr343Tyr	iucC
CP000038	3771821	T	C	1600/1743	534/580	missense variant c.1600T>C p.Phe534Leu	iucC
CP000038	3772125	C	T	165/1338	55/445	synonymous variant c.165C>T p.Thr55Thr	iucD
CP000038	3772596	C	T	636/1338	212/445	synonymous variant c.636C>T p.Asn212Asn	iucD
CP000038	3775075	A	T	1775/2199	592/732	missense variant c.1775A>T p.Tyr592Phe	iutA
CP000038	3775436	T	C	2136/2199	712/732	synonymous variant c.2136T>C p.Ala712Ala	iutA
CP000038	3774298	A	C	998/2199	333/732	missense variant c.998A>C p.Gln333Pro	iutA
CP000038	1360466	C	T	244/972	82/323	missense variant c.244C>T p.Arg82Cys	msbB
CP000038	695340	C	T	1097/3387	366/1128	missense variant c.1097C>T p.Thr366Met	rhsA
CP000038	695275	T	C	1032/3387	344/1128	synonymous variant c.1032T>C p.Ser344Ser	rhsA
CP000038	697013	C	T	2770/3387	924/1128	missense variant c.2770C>T p.His924Tyr	rhsA
CP000038	697213	G	A	2970/3387	990/1128	synonymous variant c.2970G>A p.Arg990Arg	rhsA
CP000038	4005390	G	A	2435/2949	812/982	missense variant c.2435C>T p.Pro812Leu	rhsB
CP000038	4005407	T	C	2418/2949	806/982	synonymous variant c.2418A>G p.Arg806Arg	rhsB
CP000038	4005647	C	T	2178/2949	726/982	synonymous variant c.2178G>A p.Val726Val	rhsB
CP000038	4005017	T	A	2808/2949	936/982	missense variant c.2808A>T p.Gln936His	rhsB
CP000038	4005414	T	A	2411/2949	804/982	missense variant c.2411A>T p.Gln804Leu	rhsB
CP000038	4005067	C	T	2758/2949	920/982	missense variant c.2758G>A p.Val920Ile	rhsB
CP000038	4007773	C	T	52/2949	18/982	missense variant c.52G>A p.Val18Met	rhsB

CP000038	4005133	C	A	2692/2949	898/982	missense variant c.2692G>T p.Gly898Trp	rhsB
CP000038	4005139	G	T	2686/2949	896/982	missense variant c.2686C>A p.Pro896Thr	rhsB
CP000038	4005173	A	C	2652/2949	884/982	synonymous variant c.2652T>G p.Pro884Pro	rhsB
CP000038	2824864	C	T	726/1176	242/391	synonymous variant c.726C>T p.Thr242Thr	senB
CP000038	2824653	G	A	515/1176	172/391	missense variant c.515G>A p.Ser172Asn	senB
CP000038	2824603	T	G	465/1176	155/391	missense variant c.465T>G p.Phe155Leu	senB
CP000038	2824791	C	A	653/1176	218/391	missense variant c.653C>A p.Thr218Lys	senB
CP000038	2824287	T	G	149/1176	50/391	missense variant c.149T>G p.Val50Gly	senB
CP000038	2824720	G	A	582/1176	194/391	synonymous variant c.582G>A p.Thr194Thr	senB
CP000038	2824514	T	C	376/1176	126/391	missense variant c.376T>C p.Phe126Leu	senB
CP000038	2825290	G	A	1152/1176	384/391	synonymous variant c.1152G>A p.Leu384Leu	senB
CP000038	3392326	G	A	1493/3858	498/1285	missense variant c.1493C>T p.Thr498Ile	sigA
CP000038	3391158	A	G	2661/3858	887/1285	synonymous variant c.2661T>C p.Thr887Thr	sigA
CP000038	3390404	C	T	3415/3858	1139/1285	missense variant c.3415G>A p.Glu1139Lys	sigA
CP000038	3390775	C	T	3044/3858	1015/1285	missense variant c.3044G>A p.Gly1015Glu	sigA
CP000038	3390506	C	T	3313/3858	1105/1285	missense variant c.3313G>A p.Asp1105Asn	sigA
CP000038	3390090	A	C	3729/3858	1243/1285	missense variant c.3729T>G p.Ile1243Met	sigA
CP000038	3392008	A	G	1811/3858	604/1285	missense variant c.1811T>C p.Ile604Thr	sigA
CP000038	3390667	C	T	3152/3858	1051/1285	missense variant c.3152G>A p.Gly1051Asp	sigA
CP000038	3390734	G	A	3085/3858	1029/1285	missense variant c.3085C>T p.Arg1029Cys	sigA
CP000038	3390781	C	G	3038/3858	1013/1285	missense variant c.3038G>C p.Arg1013Pro	sigA
CP000038	3392922	A	C	897/3858	299/1285	missense variant c.897T>G p.Asn299Lys	sigA
CP000038	3391874	C	A	1945/3858	649/1285	missense variant c.1945G>T p.Val649Phe	sigA
CP000038	3391963	A	T	1856/3858	619/1285	missense variant c.1856T>A p.Phe619Tyr	sigA
CP000038	3390775	C	A	3044/3858	1015/1285	missense variant c.3044G>T p.Gly1015Val	sigA
CP000038	3392033	T	G	1786/3858	596/1285	missense variant c.1786A>C p.Thr596Pro	sigA
CP000038	1857399	C	T	152/915	51/304	missense variant c.152C>T p.Ala51Val	sitA
CP000038	1857251	C	T	4/915	2/304	missense variant c.4C>T p.His2Tyr	sitA
CP000038	1857319	A	G	72/915	24/304	synonymous variant c.72A>G p.Ala24Ala	sitA
CP000038	1857367	G	A	120/915	40/304	synonymous variant c.120G>A p.Ala40Ala	sitA

CP000038	1857424	T	C	177/915	59/304	synonymous variant c.177T>C p.Pro59Pro	sitA
CP000038	1857547	C	T	300/915	100/304	synonymous variant c.300C>T p.Leu100Leu	sitA
CP000038	1857601	C	T	354/915	118/304	synonymous variant c.354C>T p.Thr118Thr	sitA
CP000038	1857607	A	C	360/915	120/304	synonymous variant c.360A>C p.Gly120Gly	sitA
CP000038	1857628	T	C	381/915	127/304	synonymous variant c.381T>C p.Asn127Asn	sitA
CP000038	1857637	A	C	390/915	130/304	synonymous variant c.390A>C p.Ala130Ala	sitA
CP000038	1858030	A	G	783/915	261/304	synonymous variant c.783A>G p.Ala261Ala	sitA
CP000038	1858045	G	A	798/915	266/304	synonymous variant c.798G>A p.Val266Val	sitA
CP000038	1858105	T	G	858/915	286/304	synonymous variant c.858T>G p.Leu286Leu	sitA
CP000038	1857811	G	A	564/915	188/304	synonymous variant c.564G>A p.Gln188Gln	sitA
CP000038	1858003	G	A	756/915	252/304	synonymous variant c.756G>A p.Ala252Ala	sitA
CP000038	1858176	A	G	15/828	5/275	synonymous variant c.15A>G p.Ala5Ala	sitB
CP000038	1858449	G	A	288/828	96/275	synonymous variant c.288G>A p.Leu96Leu	sitB
CP000038	1858485	T	C	324/828	108/275	synonymous variant c.324T>C p.His108His	sitB
CP000038	1858575	T	A	414/828	138/275	synonymous variant c.414T>A p.Arg138Arg	sitB
CP000038	1858740	A	G	579/828	193/275	synonymous variant c.579A>G p.Lys193Lys	sitB
CP000038	1858907	A	G	746/828	249/275	missense variant c.746A>G p.Asp249Gly	sitB
CP000038	1858940	G	A	779/828	260/275	missense variant c.779G>A p.Arg260His	sitB
CP000038	1858334	G	A	173/828	58/275	missense variant c.173G>A p.Arg58His	sitB
CP000038	1859048	A	G	63/858	21/285	synonymous variant c.63A>G p.Ala21Ala	sitC
CP000038	1859243	C	T	258/858	86/285	synonymous variant c.258C>T p.Thr86Thr	sitC
CP000038	1859282	G	C	297/858	99/285	synonymous variant c.297G>C p.Ser99Ser	sitC
CP000038	1859495	A	G	510/858	170/285	synonymous variant c.510A>G p.Leu170Leu	sitC
CP000038	1859537	G	A	552/858	184/285	synonymous variant c.552G>A p.Ser184Ser	sitC
CP000038	1859651	A	G	666/858	222/285	missense variant c.666A>G p.Ile222Met	sitC
CP000038	1859792	A	G	807/858	269/285	synonymous variant c.807A>G p.Gly269Gly	sitC
CP000038	1859881	G	A	42/858	14/285	missense variant c.42G>A p.Met14Ile	sitD
CP000038	1860291	C	T	452/858	151/285	missense variant c.452C>T p.Ala151Val	sitD
CP000038	1859874	C	T	35/858	12/285	missense variant c.35C>T p.Thr12Ile	sitD
CP000038	1860011	A	G	172/858	58/285	missense variant c.172A>G p.Thr58Ala	sitD

CP000038	1860023	G	A	184/858	62/285	missense variant c.184G>A p.Gly62Arg	sitD
CP000038	1860121	T	G	282/858	94/285	synonymous variant c.282T>G p.Val94Val	sitD
CP000038	1860176	A	G	337/858	113/285	missense variant c.337A>G p.Ile113Val	sitD
CP000038	1860227	A	G	388/858	130/285	missense variant c.388A>G p.Ile130Val	sitD
CP000038	1860364	G	T	525/858	175/285	missense variant c.525G>T p.Trp175Cys	sitD
CP000038	1860421	A	G	582/858	194/285	synonymous variant c.582A>G p.Ala194Ala	sitD
CP000038	1860439	T	C	600/858	200/285	synonymous variant c.600T>C p.Ser200Ser	sitD
CP000038	1860472	A	G	633/858	211/285	synonymous variant c.633A>G p.Ala211Ala	sitD
CP000038	1860516	C	T	677/858	226/285	missense variant c.677C>T p.Thr226Ile	sitD
CP000038	1860554	C	T	715/858	239/285	synonymous variant c.715C>T p.Leu239Leu	sitD
CP000038	1860574	C	T	735/858	245/285	synonymous variant c.735C>T p.Ser245Ser	sitD
CP000038	1860610	C	T	771/858	257/285	synonymous variant c.771C>T p.Val257Val	sitD
CP000038	1860628	T	C	789/858	263/285	synonymous variant c.789T>C p.Phe263Phe	sitD
CP000038	1860665	G	A	826/858	276/285	missense variant c.826G>A p.Ala276Thr	sitD
CP000038	280299	T	C	1490/2142	497/713	missense variant c.1490T>C p.Val497Ala	vgrG
CP000038	280455	T	A	1646/2142	549/713	missense variant c.1646T>A p.Leu549Gln	vgrG
CP000038	280453	C	T	1644/2142	548/713	synonymous variant c.1644C>T p.Asn548Asn	vgrG
CP000038	278875	C	T	66/2142	22/713	synonymous variant c.66C>T p.Val22Val	vgrG
CP000038	278885	C	T	76/2142	26/713	synonymous variant c.76C>T p.Leu26Leu	vgrG
CP000038	278833	G	A	24/2142	8/713	synonymous variant c.24G>A p.Thr8Thr	vgrG
CP000038	279887	C	A	1078/2142	360/713	missense variant c.1078C>A p.Leu360Met	vgrG
CP000038	278920	G	T	111/2142	37/713	missense variant c.111G>T p.Glu37Asp	vgrG
CP000038	279688	T	G	879/2142	293/713	synonymous variant c.879T>G p.Ser293Ser	vgrG
CP000038	280129	G	A	1320/2142	440/713	synonymous variant c.1320G>A p.Pro440Pro	vgrG
CP000038	280622	A	C	1813/2142	605/713	missense variant c.1813A>C p.Lys605Gln	vgrG

Table S7. The accessory genes identified associated with cef^{R}azi^{R} isolates especially the outbreak strains(Chi-squared test, no adjusted)

Gene	cef^{R}azi^{R} Presence	non-cef^{R}azi^{R} Presence	cef^{R}azi^{R} Absence	non-cef^{R}azi^{R} Absence	cef^{R}azi^{R} Presence ratio	non-cef^{R}azi^{R} Presence ratio	chisq[a]	P value	COG	Code	Product	Gene in COG
ssb_1	183	0	32	247	0.851162791	0	344.585536	6.4E-77	COG0629	L	Single-stranded DNA-binding protein	Ssb
group_5247	186	3	29	244	0.865116279	0.012145749	342.436568	1.88E-76	-	-	hypothetical protein	-
group_5236	184	2	31	245	0.855813953	0.008097166	339.929207	6.61E-76	-	-	hypothetical protein	-
group_6817	191	10	24	237	0.888372093	0.04048583	332.773297	2.39E-74	-	-	hypothetical protein	-
group_5217	185	5	30	242	0.860465116	0.020242915	331.691834	4.11E-74	-	-	hypothetical protein	-
group_6704	188	8	27	239	0.874418605	0.032388664	330.214428	8.63E-74	-	-	hypothetical protein	-
group_3220	185	6	30	241	0.860465116	0.024291498	327.972445	2.66E-73	-	-	hypothetical protein	-
group_5201	188	10	27	237	0.874418605	0.04048583	323.018842	3.19E-72	COG4974	L	Site-specific recombinase XerD	XerD
group_295	178	2	37	245	0.827906977	0.008097166	321.411221	7.14E-72	-	-	hypothetical protein	-
group_3047	184	7	31	240	0.855813953	0.028340081	321.148028	8.14E-72	-	-	hypothetical protein	-
group_4095	191	14	24	233	0.888372093	0.056680162	318.757645	2.7E-71	COG1734	K	RNA polymerase-binding transcription	DksA
group_6792	190	14	25	233	0.88372093	0.056680162	315.501114	1.38E-70	-	-	hypothetical protein	-
group_6807	191	17	24	230	0.888372093	0.068825911	308.606271	4.39E-69	-	-	hypothetical protein	-
group_5135	213	43	2	204	0.990697674	0.174089069	306.943882	1.01E-68	-	-	hypothetical protein	-
group_6635	213	43	2	204	0.990697674	0.174089069	306.943882	1.01E-68	COG1309	K	DNA-binding protein, AcrR family, includes nucleoid occlusion protein SlmA	AcrR
tap_2	213	43	2	204	0.990697674	0.174089069	306.943882	1.01E-68	COG0738	G	Fucose permease	FucP
group_5248	191	18	24	229	0.888372093	0.072874494	305.288533	2.32E-68	-	-	hypothetical protein	-
group_4105	175	6	40	241	0.813953488	0.024291498	297.493233	1.16E-66	-	-	hypothetical protein	-
group_2020	185	15	30	232	0.860465116	0.060728745	296.21192	2.2E-66	-	-	hypothetical protein	-
rop_1	174	6	41	241	0.809302326	0.024291498	294.564694	5.03E-66	-	-	hypothetical protein	-
group_6829	173	6	42	241	0.804651163	0.024291498	291.656947	2.17E-65	-	-	hypothetical protein	-
group_1922	181	13	34	234	0.841860465	0.052631579	290.700848	3.5E-65	-	-	hypothetical protein	-
group_6815	191	23	24	224	0.888372093	0.093117409	289.17475	7.52E-65	-	-	hypothetical protein	-
group_6816	190	23	25	224	0.88372093	0.093117409	285.97218	3.75E-64	-	-	hypothetical protein	-
group_6739	188	21	27	226	0.874418605	0.085020243	285.958852	3.78E-64	-	-	hypothetical protein	-
group_3077	199	34	16	213	0.925581395	0.137651822	282.326951	2.34E-63	COG1319	C	Aldehyde, CO, or xanthine dehydrogenase, FAD-binding subunit	CutB
group_4097	191	26	24	221	0.888372093	0.105263158	279.870934	8.01E-63	-	-	hypothetical protein	-
group_6740	186	21	29	226	0.865116279	0.085020243	279.708804	8.69E-63	-	-	hypothetical protein	-
group_5193	183	20	32	227	0.851162791	0.08097166	273.690111	1.78E-61	-	-	hypothetical protein	-
group_5250	191	29	24	218	0.888372093	0.117408907	270.827061	7.49E-61	-	-	hypothetical protein	-
group_6798	191	29	24	218	0.888372093	0.117408907	270.827061	7.49E-61	-	-	hypothetical protein	-
group_6799	191	29	24	218	0.888372093	0.117408907	270.827061	7.49E-61	-	-	hypothetical protein	-
group_797	185	23	30	224	0.860465116	0.093117409	270.350357	9.52E-61	-	-	hypothetical protein	-
group_6796	191	30	24	217	0.888372093	0.12145749	267.868411	3.31E-60	-	-	hypothetical protein	-
group_6797	191	30	24	217	0.888372093	0.12145749	267.868411	3.31E-60	-	-	hypothetical protein	-
group_6800	190	29	25	218	0.88372093	0.117408907	267.666363	3.66E-60	-	-	hypothetical protein	-
group_6801	190	29	25	218	0.88372093	0.117408907	267.666363	3.66E-60	-	-	hypothetical protein	-
group_5252	191	31	24	216	0.888372093	0.125506073	264.937134	1.44E-59	-	-	hypothetical protein	-
group_4093	191	33	24	214	0.888372093	0.133603239	259.155264	2.62E-58	-	-	hypothetical protein	-
group_4094	191	33	24	214	0.888372093	0.133603239	259.155264	2.62E-58	-	-	hypothetical protein	-
group_6813	191	33	24	214	0.888372093	0.133603239	259.155264	2.62E-58	-	-	hypothetical protein	-
group_3080	191	34	24	213	0.888372093	0.137651822	256.303973	1.1E-57	-	-	hypothetical protein	-
group_3134	191	34	24	213	0.888372093	0.137651822	256.303973	1.1E-57	-	-	hypothetical protein	-
group_4096	191	34	24	213	0.888372093	0.137651822	256.303973	1.1E-57	-	-	hypothetical protein	-
group_4100	191	34	24	213	0.888372093	0.137651822	256.303973	1.1E-57	-	-	hypothetical protein	-
group_5249	191	34	24	213	0.888372093	0.137651822	256.303973	1.1E-57	-	-	hypothetical protein	-
group_5251	191	34	24	213	0.888372093	0.137651822	256.303973	1.1E-57	-	-	hypothetical protein	-
group_5253	191	34	24	213	0.888372093	0.137651822	256.303973	1.1E-57	-	-	hypothetical protein	-
group_5254	191	34	24	213	0.888372093	0.137651822	256.303973	1.1E-57	-	-	hypothetical protein	-
group_5255	191	34	24	213	0.888372093	0.137651822	256.303973	1.1E-57	-	-	hypothetical protein	-
group_5256	191	34	24	213	0.888372093	0.137651822	256.303973	1.1E-57	COG3941	X	Phage tail tape-measure protein, controls tail length	HI1514
group_5257	191	34	24	213	0.888372093	0.137651822	256.303973	1.1E-57	-	-	hypothetical protein	-
group_5258	191	34	24	213	0.888372093	0.137651822	256.303973	1.1E-57	-	-	hypothetical protein	-
group_5398	191	34	24	213	0.888372093	0.137651822	256.303973	1.1E-57	-	-	hypothetical protein	-
group_5561	191	34	24	213	0.888372093	0.137651822	256.303973	1.1E-57	-	-	hypothetical protein	-
group_6793	191	34	24	213	0.888372093	0.137651822	256.303973	1.1E-57	-	-	hypothetical protein	-
group_6794	191	34	24	213	0.888372093	0.137651822	256.303973	1.1E-57	-	-	hypothetical protein	-
group_6795	191	34	24	213	0.888372093	0.137651822	256.303973	1.1E-57	-	-	hypothetical protein	-
group_6802	191	34	24	213	0.888372093	0.137651822	256.303973	1.1E-57	-	-	hypothetical protein	-
group_6803	191	34	24	213	0.888372093	0.137651822	256.303973	1.1E-57	COG3772	M	Phage-related lysozyme (muramidase), GH24 family	RrrD
group_6805	191	34	24	213	0.888372093	0.137651822	256.303973	1.1E-57	COG0735	P	Fe2+ or Zn2+ uptake regulation protein	Fur
group_6806	191	34	24	213	0.888372093	0.137651822	256.303973	1.1E-57	-	-	hypothetical protein	-
group_6808	191	34	24	213	0.888372093	0.137651822	256.303973	1.1E-57	-	-	hypothetical protein	-
group_6809	191	34	24	213	0.888372093	0.137651822	256.303973	1.1E-57	-	-	hypothetical protein	-
group_6810	191	34	24	213	0.888372093	0.137651822	256.303973	1.1E-57	-	-	hypothetical protein	-
group_6811	191	34	24	213	0.888372093	0.137651822	256.303973	1.1E-57	-	-	hypothetical protein	-
group_6812	191	34	24	213	0.888372093	0.137651822	256.303973	1.1E-57	-	-	hypothetical protein	-
group_6814	191	34	24	213	0.888372093	0.137651822	256.303973	1.1E-57	-	-	hypothetical protein	-
group_6818	191	34	24	213	0.888372093	0.137651822	256.303973	1.1E-57	-	-	hypothetical protein	-
yokD	202	48	13	199	0.939534884	0.194331984	254.077167	3.35E-57	COG2746	V	Aminoglycoside N3'-acetyltransferase	YokD
group_3079	190	34	25	213	0.88372093	0.137651822	253.181225	5.26E-57	-	-	hypothetical protein	-
group_6819	190	34	25	213	0.88372093	0.137651822	253.181225	5.26E-57	-	-	hypothetical protein	-
group_6820	190	34	25	213	0.88372093	0.137651822	253.181225	5.26E-57	-	-	hypothetical protein	-
group_2515	189	34	26	213	0.879069767	0.137651822	250.086255	2.49E-56	-	-	hypothetical protein	-
group_4098	189	34	26	213	0.879069767	0.137651822	250.086255	2.49E-56	-	-	hypothetical protein	-
xerC_1	186	31	29	216	0.865116279	0.125506073	249.478914	3.37E-56	-	-	hypothetical protein	-
group_5126	200	48	15	199	0.930232558	0.194331984	247.400627	9.57E-56	COG1403	V	5-methylcytosine-specific restriction endonuclease McrA	McrA
group_6621	200	48	15	199	0.930232558	0.194331984	247.400627	9.57E-56	-	-	hypothetical protein	-
tmrB	200	48	15	199	0.930232558	0.194331984	247.400627	9.57E-56	COG1936	F	Broad-specificity NMP kinase	Fap7
group_4042	182	28	33	219	0.846511628	0.113360324	246.249857	1.71E-55	-	-	hypothetical protein	-
group_2065	194	44	21	203	0.902325581	0.178137652	238.463558	8.51E-54	-	-	hypothetical protein	-
group_14	195	47	20	200	0.906976744	0.190283401	233.839696	8.67E-53	COG0451	M	Nucleoside-diphosphate-sugar epimerase	WcaG
group_2432	182	33	33	214	0.846511628	0.133603239	231.949489	2.24E-52	-	-	hypothetical protein	-
group_37	191	43	24	204	0.888372093	0.174089069	231.772336	2.45E-52	COG1802	K	DNA-binding transcriptional regulator, GntR family	GntR
ldrD_5	194	47	21	200	0.902325581	0.190283401	230.705008	4.18E-52	-	-	hypothetical protein	-
group_6690	182	35	33	212	0.846511628	0.141700405	226.422679	3.59E-51	-	-	hypothetical protein	-
group_4116	186	41	29	206	0.865116279	0.165991903	222.009426	3.3E-50	-	-	hypothetical protein	-
group_3048	185	40	30	207	0.860465116	0.16194332	221.707617	3.84E-50	-	-	hypothetical protein	-

group_5215	187	43	28	204	0.869767442	0.174089069	219.750813	1.03E-49	-	-	hypothetical protein	-
group_4039	186	45	29	202	0.865116279	0.182186235	211.717014	5.8E-48	-	-	hypothetical protein	-
group_6698	186	45	29	202	0.865116279	0.182186235	211.717014	5.8E-48	-	-	hypothetical protein	-
klcA	182	47	33	200	0.846511628	0.190283401	195.397547	2.11E-44	-	-	hypothetical protein	-

[a]One-sided chi-square tests were performed.

Table S8. The primers used in this study

Target	Primer sequence (5' to 3')	Amplicon size (bp)	Reference
bla$_{CTX-M-1}$ group	F: GGTTAAAAAATCACTGCGTC<br>R: TTACAAACCGTCGGTGACGA	873	Matar et al.[1]
bla$_{CTX-M-9}$ group	F: AGAGTGCAACGGATGATG<br>R: CCAGTTACAGCCCTTCGG	868	Matar et al.[1]
bla$_{CTX-M-2/8/25}$ group	F: ACCGAGCCSACGCTCAA<br>R: CCGCTGCCGGTTTTATC	221	This study
bla$_{CTX-M-14}$	F: AGCCTGCCGATCTGGTTAA<br>R: CCGGTCGTATTGCCTTTG	358	Ma et al.[2]
mphA	F: CGAAGACCTCCGAGTCCTGC<br>R: CCGCCGATACCTCCCAACT	354	Ma et al.[2]
mcr-1	F: CGGTCAGTCCGTTTGTTC<br>R: CTTGGTCGGTCTGTAGGG	309	Liu et al.[3]

Supplementary References

1 Matar, G. M. *et al.* First detection and sequence analysis of the bla-CTX-M-15 gene in Lebanese isolates of extended-spectrum-beta-lactamase-producing Shigella sonnei. *Ann Trop Med Parasitol* **101**, 511-517, doi:10.1179/136485907X193860 (2007).

2 Ma, Q. *et al.* A Waterborne Outbreak of Shigella sonnei with Resistance to Azithromycin and Third-Generation Cephalosporins in China in 2015. *Antimicrob Agents Chemother* **61**, doi:10.1128/AAC.00308-17 (2017).

3 Liu, Y. Y. *et al.* Emergence of plasmid-mediated colistin resistance mechanism MCR-1 in animals and human beings in China: a microbiological and molecular biological study. *Lancet Infect Dis* **16**, 161-168, doi:10.1016/S1473-3099(15)00424-7 (2015).

Uncropped images of Figure S1

a

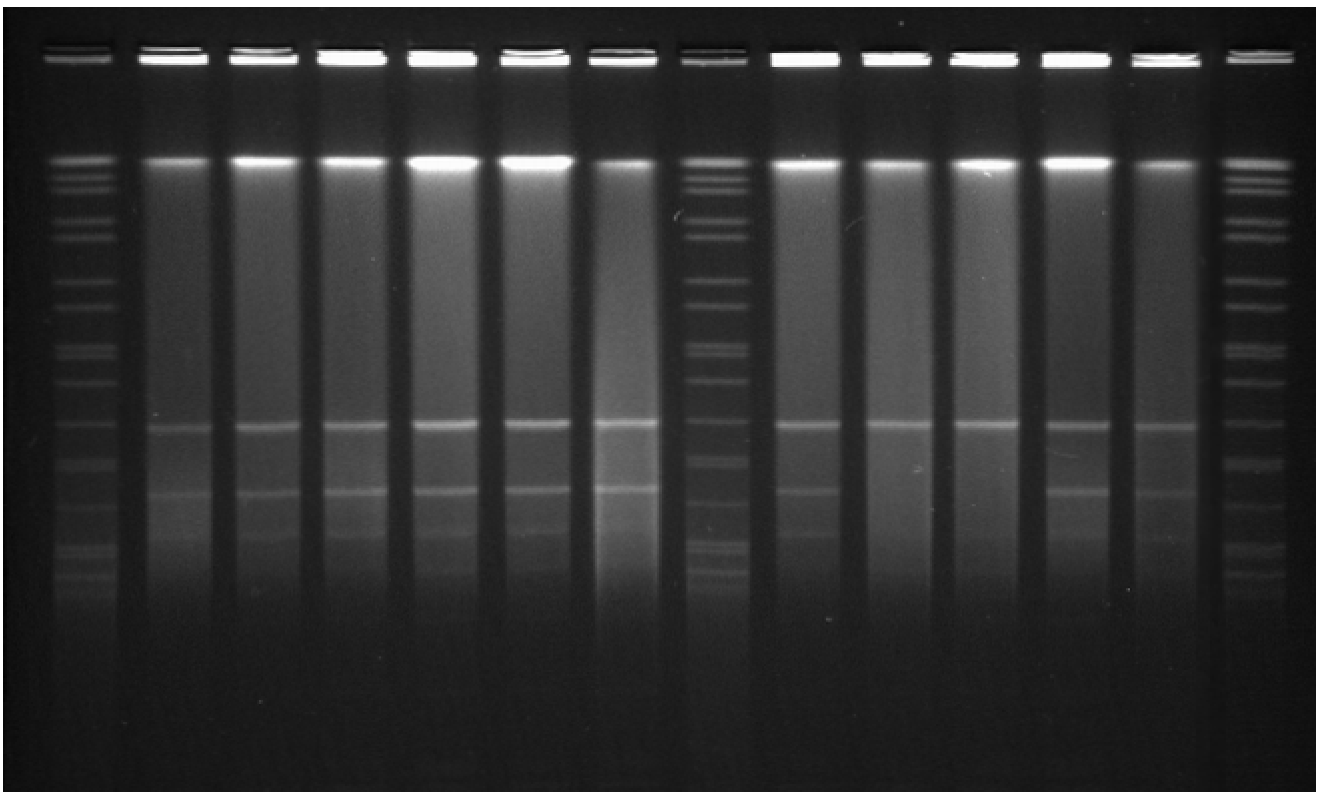

b

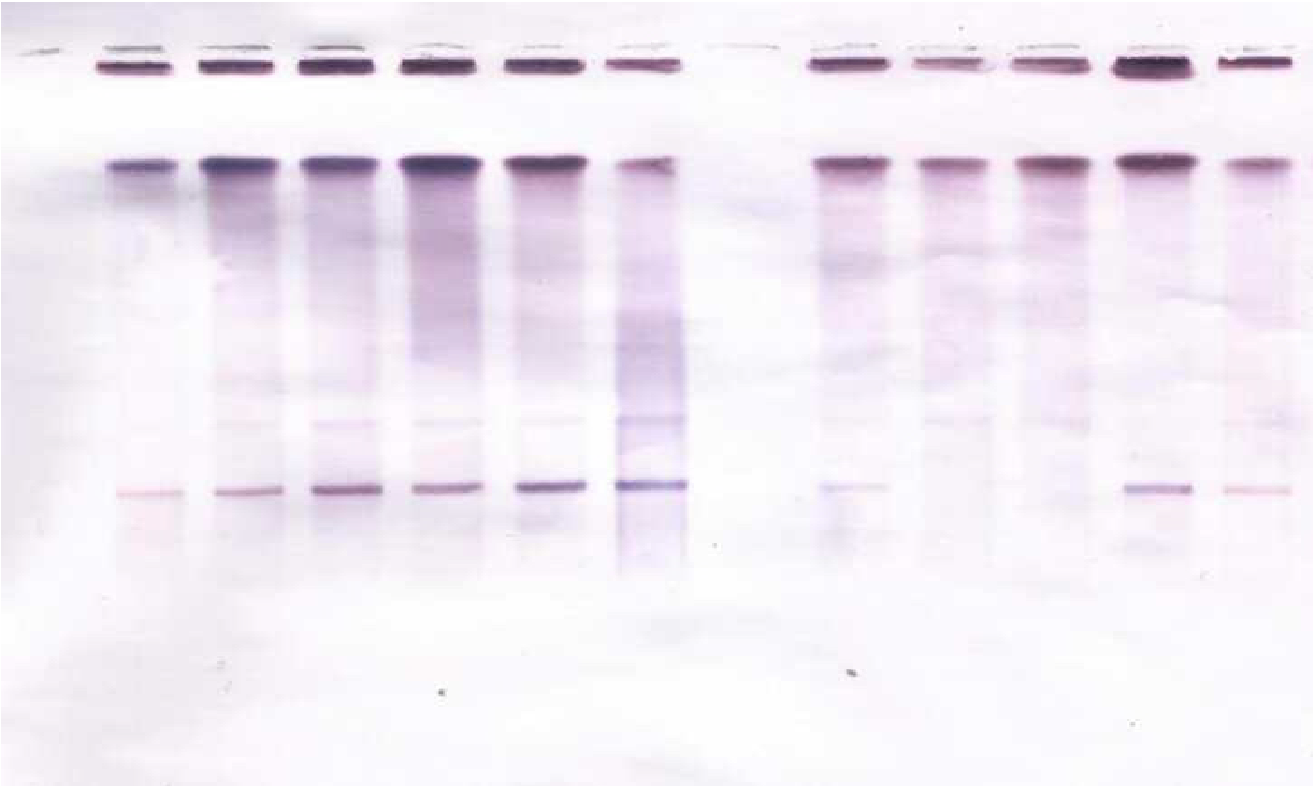

c

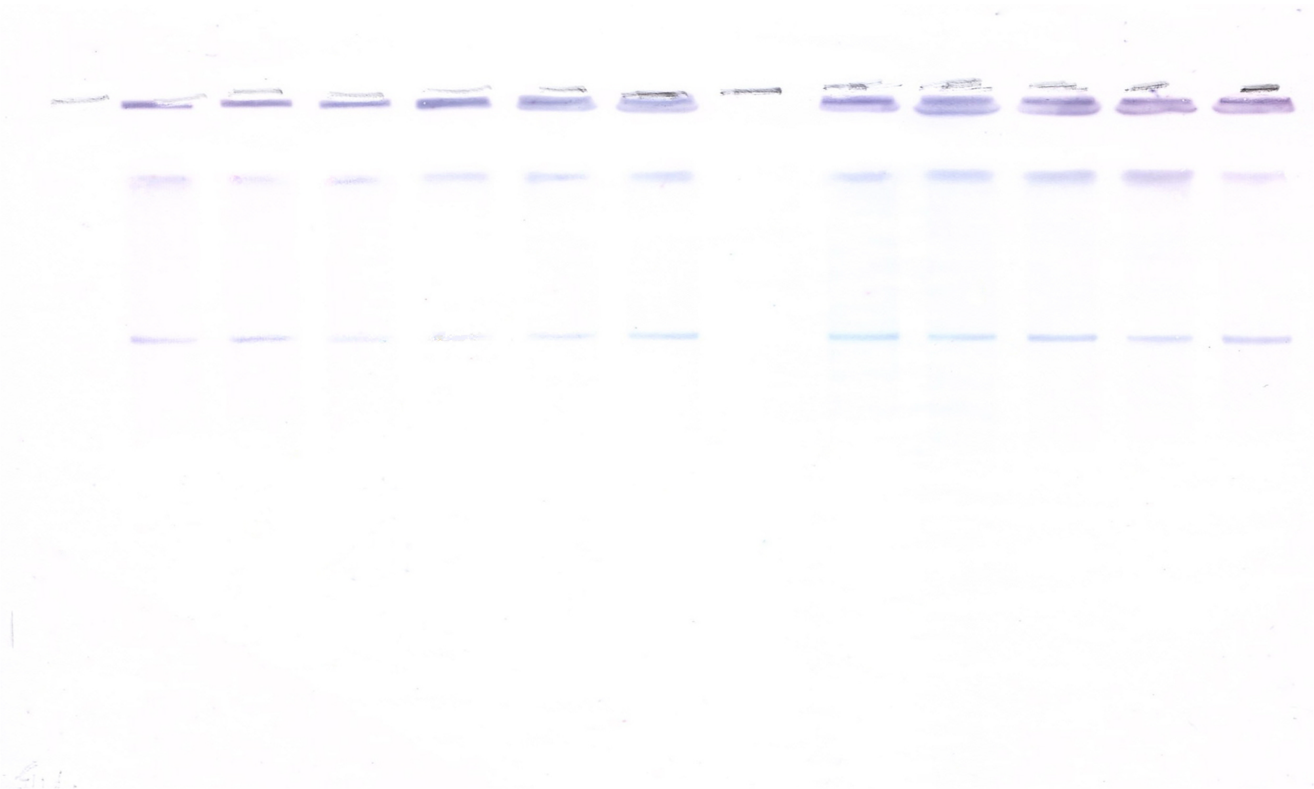

d

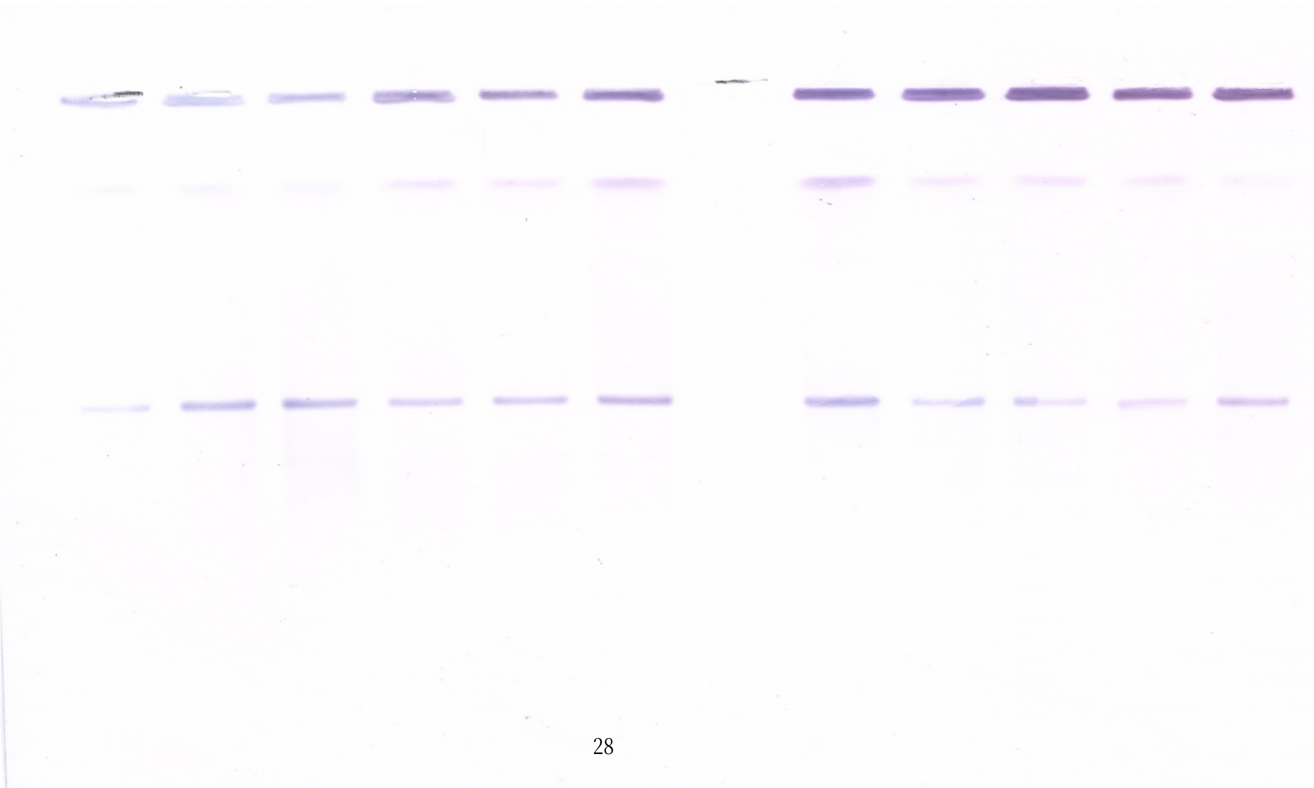

Supplement: Supplementary file 1 — Supplementary Information [file 41467_2022_35136_MOESM1_ESM.pdf]
