# Supplementary material for: A Shigella sonnei clone with extensive drug resistance associated with waterborne outbreaks in China
Source: Nat Commun. 2022 Nov 30;13:7365. doi: 10.1038/s41467-022-35136-1 (PMC9709761; doi:10.1038/s41467-022-35136-1)
Supplement: Supplementary file 3 — Description of Additional Supplementary Files [file 41467_2022_35136_MOESM3_ESM.pdf]

Table S3. The genome-sequenced *S. sonnei* isolates used in this study. Due to the page size limitation, Supplementary Table S3 is provided as a separate EXCEL file.
