# Supplementary material for: A Shigella sonnei clone with extensive drug resistance associated with waterborne outbreaks in China
Source: Nat Commun. 2022 Nov 30;13:7365. doi: 10.1038/s41467-022-35136-1 (PMC9709761; doi:10.1038/s41467-022-35136-1)
Supplement: Supplementary file 5 — Reporting Summary. [file 41467_2022_35136_MOESM5_ESM.pdf]

nature portfolio

Corresponding author(s): Hongbin Song, Xuebin Xu, and Juntao Yang

Last updated by author(s): Nov 15, 2022

# Reporting Summary

Nature Portfolio wishes to improve the reproducibility of the work that we publish. This form provides structure for consistency and transparency in reporting. For further information on Nature Portfolio policies, see our Editorial Policies and the Editorial Policy Checklist.

Please do not complete any field with "not applicable" or n/a. Refer to the help text for what text to use if an item is not relevant to your study.
For final submission: please carefully check your responses for accuracy; you will not be able to make changes later.

## Statistics

For all statistical analyses, confirm that the following items are present in the figure legend, table legend, main text, or Methods section.

| n/a | Confirmed | |
|---|---|---|
| ☐ | ☒ | The exact sample size (*n*) for each experimental group/condition, given as a discrete number and unit of measurement |
| ☒ | ☐ | A statement on whether measurements were taken from distinct samples or whether the same sample was measured repeatedly |
| ☐ | ☒ | The statistical test(s) used AND whether they are one- or two-sided<br>*Only common tests should be described solely by name; describe more complex techniques in the Methods section.* |
| ☒ | ☐ | A description of all covariates tested |
| ☒ | ☐ | A description of any assumptions or corrections, such as tests of normality and adjustment for multiple comparisons |
| ☐ | ☒ | A full description of the statistical parameters including central tendency (e.g. means) or other basic estimates (e.g. regression coefficient) AND variation (e.g. standard deviation) or associated estimates of uncertainty (e.g. confidence intervals) |
| ☐ | ☒ | For null hypothesis testing, the test statistic (e.g. *F*, *t*, *r*) with confidence intervals, effect sizes, degrees of freedom and *P* value noted<br>*Give P values as exact values whenever suitable.* |
| ☐ | ☒ | For Bayesian analysis, information on the choice of priors and Markov chain Monte Carlo settings |
| ☒ | ☐ | For hierarchical and complex designs, identification of the appropriate level for tests and full reporting of outcomes |
| ☒ | ☐ | Estimates of effect sizes (e.g. Cohen's *d*, Pearson's *r*), indicating how they were calculated |

*Our web collection on statistics for biologists contains articles on many of the points above.*

## Software and code

Policy information about availability of computer code

| | |
|---|---|
| Data collection | Illumina Sequencing Platform (Hiseq, Miseq). Oxford Nanopore Sequencing platform (MinION). |
| Data analysis | The following software was used: Trimmomatic v0.39, Spades v3.15.2, Canu v1.6, Pilon v1.24, RedDog v1beta.10.3, FastTree v2.1.8, BEAST2 v2.6.6, Mykrobe v0.12.1, MLST tool v2.19.0, RGI v5.1.1, Prokka v1.14.6, PlasmidFinder v2.0.1, ISfinder, ResFinder v4.0.1, BLAST v2.12.0, Roary v3.13.0, Plink v1.07, and R v4.02.<br><br>The code that supports the findings of this study is available from the corresponding authors on request. Code requestors will be required to sign a code access agreement in accordance with the confidentiality requirements of the author's affiliations. |

For manuscripts utilizing custom algorithms or software that are central to the research but not yet described in published literature, software must be made available to editors and reviewers. We strongly encourage code deposition in a community repository (e.g. GitHub). See the Nature Portfolio guidelines for submitting code & software for further information.

# Data

Policy information about availability of data

All manuscripts must include a data availability statement. This statement should provide the following information, where applicable:

- Accession codes, unique identifiers, or web links for publicly available datasets
- A description of any restrictions on data availability
- For clinical datasets or third party data, please ensure that the statement adheres to our policy

The publicly available sequences used in this study are available in GenBank under accession numbers KX008967 [https://www.ncbi.nlm.nih.gov/nuccore/KX008967.1/], KY471628 [https://www.ncbi.nlm.nih.gov/nuccore/KY471628.1/], KP347127 [https://www.ncbi.nlm.nih.gov/nuccore/KP347127.1/], LN624486 [https://www.ncbi.nlm.nih.gov/nuccore/LN624486.1/], LC056425 [https://www.ncbi.nlm.nih.gov/nuccore/LC056425.1/], and NC_007384 [https://www.ncbi.nlm.nih.gov/nuccore/NC_007384.1/]. The sequencing data generated in this study have been deposited in NCBI Sequence Read Archive under the BioProject number PRJNA835603 [https://www.ncbi.nlm.nih.gov/biosample/?LinkName=bioproject_biosample_all&from_uid=835603]. The annotated plasmid sequences in this study have been deposited in GenBank under accession numbers MG299127 [https://www.ncbi.nlm.nih.gov/nuccore/MG299127.1/]-MG299153 [https://www.ncbi.nlm.nih.gov/nuccore/MG299153.1/] and ON461899 [https://www.ncbi.nlm.nih.gov/nuccore/ON461899.1/]-ON461902 [https://www.ncbi.nlm.nih.gov/nuccore/ON461902.1/]. Source data are provided with this paper.

# Human research participants

Policy information about studies involving human research participants and Sex and Gender in Research.

| | |
|---|---|
| Reporting on sex and gender | Our study does not include sex- or gender-based analyses. Furthermore, there is no evidence that the strains in our study are associated with sex or gender. |
| Population characteristics | The majority (92.4%) of the 433 patients in our study were under 14 years old, and male patients accounted for 48.3%. They all showed symptoms of diarrhea, followed by fever (94.0%), abdominal pain (70.2%), vomiting (67.7%), rectal tenesmus (50.3%), nausea (22.2%), and dizziness (1.6%). All these patients recovered, with no deaths. |
| Recruitment | In this study, we investigated 307 Chinese Shigella sonnei strains, including 155 outbreak strains and 152 sporadic strains. The 155 outbreak strains were recovered from samples of 433 patients, who were the entire population of patients in the six outbreaks. The 152 sporadic strains were recovered from samples of 152 patients, who were randomly sampled from historical sporadic patients in our surveillance system.<br><br>Since our study focused on patients and strains from only six outbreaks, there may be selection bias. Outbreak strains tend to have a similar profile of resistance to multiple antibiotics, which may lead to an overestimation of the number of resistance genes carried by these strains. |
| Ethics oversight | The study was approved by the review board of the Chinese PLA CDC, Beijing, China (No. #20220523). All patients provided informed consent for the collection of samples and relevant information. |

Note that full information on the approval of the study protocol must also be provided in the manuscript.

# Field-specific reporting

Please select the one below that is the best fit for your research. If you are not sure, read the appropriate sections before making your selection.

☒ Life sciences ☐ Behavioural & social sciences ☐ Ecological, evolutionary & environmental sciences

# Life sciences study design

All studies must disclose on these points even when the disclosure is negative.

| | |
|---|---|
| Sample size | In this study, we investigated 307 Chinese Shigella sonnei strains, including 155 outbreak strains and 152 sporadic strains. The 155 outbreak strains were recovered from samples of 433 patients, who were the entire population of patients in the six outbreaks. The 152 sporadic strains were recovered from samples of 152 patients; the number of sporadic strains was determined for comparability with the number of outbreak strains. Based on previous literature, this sample size is sufficient. |
| Data exclusions | One strain was excluded due to low mapping rates. |
| Replication | All experiments were repeated twice or more, and reproducibility was confirmed. |
| Randomization | The samples were not allocated into different groups in this study. Therefore, randomization is not relevant. |

Blinding | In all experiments, investigators were blinded to minimize their impacts on experiments.

# Reporting for specific materials, systems and methods

We require information from authors about some types of materials, experimental systems and methods used in many studies. Here, indicate whether each material, system or method listed is relevant to your study. If you are not sure if a list item applies to your research, read the appropriate section before selecting a response.

## Materials & experimental systems

| n/a | Involved in the study |
|---|---|
| ☒ | ☐ Antibodies |
| ☒ | ☐ Eukaryotic cell lines |
| ☒ | ☐ Palaeontology and archaeology |
| ☒ | ☐ Animals and other organisms |
| ☒ | ☐ Clinical data |
| ☒ | ☐ Dual use research of concern |

## Methods

| n/a | Involved in the study |
|---|---|
| ☒ | ☐ ChIP-seq |
| ☒ | ☐ Flow cytometry |
| ☒ | ☐ MRI-based neuroimaging |

This checklist template is licensed under a Creative Commons Attribution 4.0 International License, which permits use, sharing, adaptation, distribution and reproduction in any medium or format, as long as you give appropriate credit to the original author(s) and the source, provide a link to the Creative Commons license, and indicate if changes were made. The images or other third party material in this article are included in the article's Creative Commons license, unless indicated otherwise in a credit line to the material. If material is not included in the article's Creative Commons license and your intended use is not permitted by statutory regulation or exceeds the permitted use, you will need to obtain permission directly from the copyright holder. To view a copy of this license, visit http://creativecommons.org/licenses/by/4.0/
